# Circular DNA Enrichment Sequencing (CIDER-Seq) Facilitated the Discovery of a New World (NW) Begomovirus–Alphasatellite Complex Associated with Leaf Curl Disease of Tomato in Morelos, Mexico

**DOI:** 10.3390/v18090950

**Published:** 2026-08-30

**Authors:** Enrique Alejandro Guevara-Rivera, Edgar Antonio Rodríguez-Negrete, Karina Atriztán-Hernández, Beatríz Eugenia Jiménez-Moraila, Guillermo Corona-Armenta, Norma Elena Leyva-López, Marianne Lizbeth Mireles-Varela, Eduardo Rodríguez-Bejarano, Rosa Lozano-Durán, Jesús Méndez-Lozano

**Affiliations:** 1 Departamento de Biotecnología Agrícola, CIIDIR-Unidad Sinaloa, Instituto Politécnico Nacional, Guasave 81101, Sinaloa, Mexico; enrique95alejandro@hotmail.com (E.A.G.-R.); erodriguezn@ipn.mx (E.A.R.-N.); neleyval@ipn.mx (N.E.L.-L.);; 2 Laboratorio de Servicios Genómicos de la Unidad de Genómica Avanzada, Cinvestav, Km 9.6 Libramiento Norte Carretera Irapuato-León, Irapuato 36824, Guanajuato, Mexico; 3 Área de Genética, Facultad de Ciencias, Instituto de Hortofruticultura Subtropical y Mediterranea “La Mayora”(IHSM), Universidad de Málaga-Consejo Superior de Investigaciones Científicas, 29071 Malaga, Spain; edu_rodri@uma.es; 4 Department of Plant Biochemistry, Center for Plant Molecular Biology (ZMBP), Eberhard Karls University, 72076 Tübingen, Germany; rosa.lozano-duran@uni-tuebingen.de; 5 Cluster of Excellence GreenRobust, Eberhard Karls University, 72076 Tübingen, Germany

**Keywords:** CIDER-Seq, leaf curl disease of tomato, New World (NW) begomovirus, tomato-associated alphasatellites, begomovirus–alphasatellite complex

## Abstract

A circular DNA enrichment sequencing (CIDER-Seq) approach identified a New World (NW) begomovirus–alphasatellite complex associated with a leaf curl disease of tomato from plants collected in Morelos, México. Using one of 21 samples showing virosis-associated symptoms, CIDER-Seq data revealed the presence of various NW begomovirus species, namely *Begomovirus solanumaureivariati* (tomato golden mottle virus: ToGMoV), *Begomovirus solanumseveri* (tomato severe leaf curl virus: ToSLCV), and *Begomovirus solanumlapazense* (tomato chino La Paz virus: ToChLPV). Additionally, the alphasatellites *Whiflysatellite guatemalaense* (whitefly-associated Guatemala alphasatellite 1: WfaGA1) and *Clecrusatellite guatemalaense* (whitefly-associated Guatemala alphasatellite 2: WfaGA2) were identified, demonstrating that begomoviruses can co-infect with alphasatellites; however, the pathogenic implications of the alphasatellite–helper begomovirus association remain incompletely understood. PCR-based detection and Sanger sequencing of circular viral genomes revealed a high frequency of detection of mixed infections comprising all components of the begomovirus–alphasatellite complex, confirming the reliability of the CIDER-Seq approach in producing high-fidelity sequences. Molecular and biological analysis revealed the complex adaptation to tomato, the monopartite nature of ToSLCV/ToChLPV species, and the ability of these begomovirus species to act as helper viruses. To the best of our knowledge, this is the first report of leaf curl disease of tomato associated with a begomovirus–alphasatellite complex in Mexico.

## 1. Introduction

Geminiviruses are non-enveloped plant viruses characterized by circular single-stranded DNA (ssDNA) genomes that range from 2.5 to 5.2 kb, encapsidated in twined quasi-icosahedral particles. The *Geminiviridae* family is divided into 15 genera based on their host range, type of insect vector, genomic organization, and phylogenetic relationships [1]. Members of the *Begomovirus* genus are transmitted by the polyphagous whitefly (*Bemisia tabaci*), with more than 440 reported species, and represent the most successful group of geminiviruses affecting a wide range of dicotyledonous crops in tropical and subtropical producing regions worldwide [2].

Begomoviruses are classified into two groups based on genomic phylogenetic features: Old World begomoviruses (OW; Europe, Africa, Asia, Oceania and the Indian subcontinent) and New World begomoviruses (NW; Latin America and Mesoamerica) [3]. NW begomoviruses are mostly bipartite, with two genomic components referred to as DNA-A and DNA-B of similar sizes, ranging from 2.6 to 2.8 kb.

The DNA-A encodes proteins related to viral genome encapsidation (capsid protein, CP, in the virion sense) and those involved in viral replication, plant cycle regulation, transactivation of viral genes, and suppression of host defense responses (AC1/Rep, AC2/TrAP, AC3/REn, and AC4, in the complementary sense). The DNA-B encodes proteins involved in cell-to-cell and systemic viral movement (in the virion sense BV1/NSP and in the complementary sense BC1/MP).

The majority of OW begomoviruses are monopartite, with a genomic organization similar to that of the DNA-A of bipartite viruses. They also encode an additional Open Reading Frame (ORF), partially overlapping with that encoding the CP, named V2. The V2 protein is involved in viral movement and silencing suppression [4]. The genes on the virion sense and complementary sense strands are separated by an intergenic region (IR), which contains viral transcription regulatory elements and a replication origin located at a stem-loop structure that includes the nonanucleotide sequence (TAATATATTAC), conserved within the *Geminiviridae* family [5].

Begomoviruses can be found in association with additional circular ssDNA satellite molecules [6]. DNA satellites require a helper begomovirus for cellular movement, encapsidation, and transmission by the whitefly vector. Three types of DNA satellites have been described: betasatellites (only found in OW), alphasatellites (identified in both OW and NW), and deltasatellites (only found in NW) [7,8].

While betasatellites have been extensively described as pathogenicity determinants associated with various plant diseases caused by OW begomoviruses, the biological effects of alphasatellites in association with begomoviruses are poorly understood [9].

Geminivirus-associated alphasatellites (family *Alphasatellitidae*, subfamily *Gaminialphasatellinae*) are divided into nine genera with more than 40 species [10]. Alphasatellite genomes are approximately 1.4 kb, resembling the DNA-R component of nanoviruses. Their genomes display a single ORF in the virion sense, encoding a replication-associated protein named alpha-Rep, a protein that supports alphasatellite autonomous replication. While the IR of the genome includes an adenine-rich region and features a stem-loop structure that contains the replication origin with a nanovirus-related conserved nonanucleotide sequence (TAGTATTAC) [9].

Alphasatellites were first reported in association with OW monopartite begomoviruses causing cotton leaf curl disease in Pakistan, Ageratum vein disease in Southeast Asia, and tomato yellow leaf curl disease in China. Generally, they are shown to ameliorate disease symptoms and reduce viral accumulation [8,11,12].

On the other hand, in the Americas, alphasatellites have been detected in association with NW bipartite begomoviruses infecting non-cultivated plants in Brazil and Cuba [13,14], as well as crops including watermelon in Venezuela [15] and tomato in Brazil [16], and have also been detected through vector-enabled metagenomics (VEM) from viruliferous whitefly samples in Guatemala and Puerto Rico [17].

Interestingly, contrary to OW begomovirus–alphasatellite associations, NW alphasatellites interact promiscuously with different NW begomoviruses, resulting in an increase in symptom severity and interference with helper virus transmission [18].

Advances in second-generation high-throughput sequencing (HTS) technologies based on short-read sequencing (i.e., Illumina Sequencing) have facilitated the production of extensive metagenomic data about begomovirus and associated DNA satellite diversity. Although this has facilitated the identification of previously unknown viruses [17,19,20,21], accurate identification of viral and satellite DNA components remains difficult because of the risk of assembling artificial chimeric viral genomes from short sequence reads.

The established third-generation HTS technologies based on long-read sequencing (i.e., PacBio and Oxford Nanopore Sequencing) have permitted the direct sequencing of full-length molecules. For geminiviruses, a method named circular DNA enrichment sequencing (CIDER-Seq) allows for the identification of all enriched circular sequences through accurate long-read sequencing of viral-sized circular DNA molecules [22].

Tomato (*Solanum lycopersicum*), according to the Food and Agriculture Organization, is the most important vegetable crop worldwide [23]. Mexico is the ninth largest world producer, with cultivation distributed mostly in the states of Sinaloa, San Luis Potosi, and Michoacán [24].

In Mexico, the introduced OW begomovirus species *Begomovirus coheni* (tomato yellow leaf curl virus (TYLCV)) [25] and several NW begomovirus species, including *Begomovirus solanumflavi* (chino del tomate virus; CdTV) [26], *Begomovirus capsicummusivi* (pepper golden mosaic virus; PepGMV) [27], *Begomovirus capsicumhuastecoense* (pepper Huasteco yellow vein virus; PHYVV) [28], *Begomovirus solanumvariati* (tomato mottle virus; ToMoV) [29], *Begomovirus solanumlapazense* (tomato chino La Paz virus; ToChLPV), *Begomovirus solanumseveri* (tomato severe leaf curl virus; ToSLCV), and *Begomovirus solanumaureivariati* (tomato golden mottle virus; ToGMoV) [27,30,31,32], have been associated with leaf curl disease of tomato. However, to date, their association with DNA satellites has not been reported.

In the present study, using a CIDER-Seq approach followed by biological characterization, a NW begomovirus–alphasatellite complex associated with a leaf curl disease of tomato in Morelos, Mexico, is reported for the first time. The obtained results expand the knowledge of DNA viral agents associated with leaf curl disease of tomato in Mexico, highlighting the importance of applying NGS tools in order to implement viromic-based epidemiological and ecological heuristic approaches.

## 2. Materials and Methods

### 2.1. DNA Viral Species Detection and Molecular Cloning

#### 2.1.1. Plant Sampling and Begomovirus Molecular Detection

During 2018, tomato crops grown in open fields in Morelos State, Mexico, showed virus-related symptoms, including leaf curling, yellowing, plant stunting and infestation with the *Bemisia tabaci* whitefly vector, suggesting the presence of a viral etiological agent spread by whitefly. In total, 21 plant samples (A1–A21), including both leaf and fruit tissues, were collected, and total DNA was extracted using the CTAB extraction method [33].

For generalist begomovirus detection, 100 ng of DNA was used as a template for PCR molecular detection with degenerate universal primers for DNA-A [32] or DNA-B segments [34]. For DNA-B generalist detection, PCR amplicons were obtained using Phusion High-Fidelity DNA Polymerase (New England Biolabs, Ipswich, MA, USA) following the manufacturer’s instructions, resolved by electrophoresis in a 1% agarose gel, and gel-purified with the PureLink Quick Gel Extraction Kit (Thermo Fisher Scientific, Carlsbad, CA, USA). Purified fragments were cloned into the pMiniT 2.0 vector (New England Biolabs), and ligation products were transformed into DH10B *E. coli* chemically competent cells. Positive clones were selected through plasmid DNA digestion with the EcoRI restriction enzyme, and two clones per sample were fully sequenced. Begomovirus generalist primers are summarized in Table S1.

#### 2.1.2. Cloning of Full-Length Begomovirus and Alphasatellite Genomes

Full-length genome cloning of begomovirus species ToGMoV and ToSLCV was performed using the previously described protocol [35]. Total DNA (100 ng) from the randomly selected sample (A20) with used as a template for Rolling Circle Amplification (RCA) using the Illustra TempliPhi Amplification kit (Cytiva, Marlborough, MA, USA) following the manufacturer’s instructions. The resulting concatemers were digested with EcoRI or KpnI restriction enzymes, which, according to an in-silico analysis of PacBio-derived consensus sequences, linearized ToGMoV and ToSLCV DNA-A or ToGMoV DNA-B, respectively. The digestion products were resolved by electrophoresis in a 1% agarose gel, and the putative full-length monomeric components of ~2.7 kb were gel-purified with the PureLink Quick Gel Extraction Kit (Thermo Fisher Scientific). Purified fragments were cloned into the pGreen0029 vector [36], digested with the corresponding enzyme, and ligation products were transformed into DH10B *E. coli* chemically competent cells. Positive clones were selected through plasmid DNA digestion with enzymes specific to differentiate between DNA-A and DNA-B segments according to virtual maps derived from consensus sequences obtained from PacBio data.

For ToChLPV, WfaGA1, and WfaGA2, full-length genomes were obtained by PCR using adjacent primers (Table S1), including SacI or XbaI single cut sites for ToChLPV DNA-A and WfaGA1 or WfaGA2, respectively, based on an in-silico analysis of the corresponding PacBio-derived consensus sequences. Viral full-length genome amplification was performed with Phusion High-Fidelity DNA Polymerase (New England Biolabs) and 100 ng of total DNA from the selected sample (A20) as the template. PCR products were resolved by electrophoresis in a 1% agarose gel, and the putative full-length monomeric components of ~2.7 kb and ~1.3 kb for begomovirus and alphasatellite genomes, respectively, were gel-purified using the PureLink Quick Gel Extraction Kit (Thermo Fisher Scientific). Purified fragments were cloned into the pMiniT 2.0 vector (New England Biolabs), and the ligation products were transformed into DH10B *E. coli* chemically competent cells. Positive clones were selected by plasmid DNA digestion with the EcoRI restriction enzyme.

For all begomovirus and alphasatellite segments, two independent clones were fully sequenced using a primer walking strategy with both cloning vector primers (M13-F/M13-R, for pGreen0029, or SP6/T7 for pMiniT 2.0) and viral-specific primers (Table S1).

The obtained sequences were assembled using the SeqMan software (DNASTAR Inc, Madison, WI, USA, version 8.1.3.), and the identity of the sequences was verified by searching for the closest match in the NCBI core_nt database using BLAST, https://blast.ncbi.nlm.nih.gov/Blast.cgi, accessed on 9 August 2026.

#### 2.1.3. Begomovirus and Alphasatellite Infectious Clones Construction

Infectious begomovirus and alphasatellite partial dimeric clones were constructed as previously described [37]. For ToGMoV, fragments of 2.0 kb and 0.2 kb were released with HindIII/EcoRI or SacI/KpnI double digestion for DNA-A and DNA-B, respectively, from the corresponding monomeric constructs. For ToSLCV and ToChLPV, fragments of 0.3 kb and 0.5 kb were released with SacI/KpnI or SacI/XhoI double digestion of the corresponding monomeric DNA-A constructs. For WfaGA1 and WfaGA2, fragments of 0.6 kb and 1.3 kb were obtained by SacI/StyI or XbaI/SacI double digestion of the corresponding monomeric constructs. Digestion fragments were cloned into the pGreen0029 vector linearized through the corresponding compatible double digestion to yield ToGMoV-A 0.7 mer, ToGMoV-B 0.07 mer, ToSLCV-A 0.1 mer, ToChLPV-A 0.2 mer, WfaGA1 0.4 mer, and WfaGA2 0.9 mer. The resulting intermediates were linearized with EcoRI or SacI (for ToGMoV-A 0.7 mer and ToGMoV-B 0.07 mer), SacI (for ToSLCV-A 0.1 mer and ToChLPV-A 0.2 mer), and SacI or XbaI (for WfaGA1 0.4 mer and WfaGA2 0.9 mer), dephosphorylated with Shrimp Alkaline Phosphatase (rSAP) (New England Biolabs), and then the fragments were ligated with a monomeric copy of the full-length viral genome released with the same enzyme from the corresponding monomeric constructs, yielding ToGMoV-A 1.7 mer, ToGMoV-B 1.07 mer, ToSLCV-A 1.1 mer, ToChLPV-A 1.2 mer, WfaGA1 1.4 mer and WfaGA2 1.9 mer hemidimeric constructs. In each cloning step, positive clones were verified by enzymatic digestion. Positive partial dimeric clones were transformed into the GV3101 *Agrobacterium tumefaciens* strain by electroporation (25 μF, 400 Ω, 2500 V) in a Gene Pulser XCell (Bio-Rad, Hercules, CA, USA) apparatus, and the resulting transformant strains were stored at −70 °C before use.

### 2.2. Circular DNA Enrichment Sequencing (CIDER-Seq) Sample Preparation

#### 2.2.1. Circular DNA Preparation and Enrichment

For the CIDER-Seq approach, one begomovirus-positive sample (A20) was randomly selected. Sample preparation for CIDER-Seq was performed as described previously [38] with minor modifications. Briefly, for linear DNA elimination, 1 µg of total DNA was treated with 10 units of Exonuclease V (RecBCD) (New England Biolabs) at 37 °C for 30 min, followed by heat inactivation at 70 °C for 30 min. The digestion product was column-purified with the PureLink Plant Total DNA purification kit (Thermo Fisher Scientific) according to the manufacturer’s instructions. Then, 100 ng of linear-free DNA was used for circular DNA enrichment by Rolling Circle Amplification (RCA) using the Illustra TempliPhi Amplification kit (Cytiva) following the manufacturer’s instructions, and the amplified circular DNA was purified by sodium acetate/ethanol precipitation.

#### 2.2.2. Processing of Amplified DNA for Long-Read Sequencing

After the previous stage, 10 µg of amplified DNA was used in a debranching reaction with 5 U of Phi29 DNA polymerase (Thermo Fisher Scientific) without a primer at 30 °C for 2 h, which was stopped by heating at 65 °C for 2 min. The product was purified by sodium acetate/ethanol precipitation. The product was then treated with 1.25 U of S1 nuclease (New England Biolabs) at 37 °C for 30 min and purified by sodium acetate/ethanol precipitation. Then, the de-branched DNA was treated with 0.06 U of T4 DNA polymerase (Thermo Fisher Scientific) and 0.2 u of *E. coli* DNA polymerase I (New England Biolabs) in the presence of a final concentration of 0.2 mM dNTPs. The reaction was incubated at 25 °C for 1 h and stopped by heating at 75 °C for 5 min. The product was dephosphorylated by adding 5 U of Shrimp Alkaline Phosphatase (rSAP) (New England Biolabs) with incubation at 37 °C for 10 min and was then stopped by heating to 75 °C for 5 min. The repaired DNA was purified using Ampure-Xp Magnetic Beads (Sigma-Aldrich, Burlington, MA, USA) at a 1.5× volumetric ratio. The resulting purified product was stored at −20 °C before use.

### 2.3. CIDER-Seq Library Processing and Begomovirus Consensus Sequence Construction

The CIDER-Seq purified product was sent to the sequencing facilities at LabSerGene CINVESTAV (LANGEBIO, Irapuato, GTO, Mexico) and used for Single Molecule Real-Time (SMRT) sequencing with a Sequel System (Pacific Biosciences, Menlo Park, CA, USA). The resulting library was processed with CCS (Consensus Circular Sequencing, v4.0.0) using the default parameters https://github.com/PacificBiosciences/pbbioconda (accessed on 5 October 2023) to obtain high-fidelity reads (under default conditions). This resulted in high-fidelity (Hi-Fi) reads that were subjected to a deconcatamerization process using the circular DNA enrichment sequencing (CIDER-Seq 2; version 1) with the deconcat algorithm [38] to validate Hi-Fi reads corresponding to circular sequences. Only sequences with a score of 2 from the deconcat rounds were kept for further processing. Subsequently, reads were mapped to the *Solanum lycopersicum* genome sequence (GCF_000188115.5) using the program minimap2 (2.24-r1122; -ax map-bp) [39]. The resulting “.sam” files were then subjected to the view (-b, -o), sort (-o), and index (-bc) commands of Samtools (1.13) [40] to separate them based on whether they were mapped to the *S. lycopersicum* genomic sequence. The mapped sequences were considered eccDNAs originating from the tomato genome, while the unmapped sequences were identified as viral genomes. The quality of the mapping process was evaluated using qualimap_v2.1 with default conditions [41].

The viral sequences ranging from 2501 to 3000 bp were aligned, beginning with the motif “ACCGGAT” and concluding with the sequence “ATAATATT” [1] using MAFFT (v7.526) with default conditions [42]. The primary identity of the high-fidelity reads was assessed by aligning them against the reference database of viral genomic sequences (ref_viruses_rep_genomes) utilizing BLAST+ (Nucleotide-Nucleotide BLAST 2.12.0+): e-value 1 × 10^−1^, num_threads 4, outfmt 5 [43]. All sequences without an assigned identity were submitted to the BLAST of the NCBI “https://blast.ncbi.nlm.nih.gov/Blast.cgi (accessed on 10 November 2024) using the core_nt database, and the sequences that remained without identity after all the prior steps were labeled “no hit.” Finally, an in-house built R (version 4.3.3; RStudio 2025.05.1 + 513) script with the “consensus” function from the seqinr library, using the majority method, was used alongside the alignments of each cognate to generate a single consensus sequence. Subsequently, the percentage similarity of each consensus sequence was compared against the full-length genomes obtained through Sanger sequencing, corresponding to the same species and/or cognate. Furthermore, open reading frames (ORFs) in the consensus were examined using SnapGene software (GLS Biotech, LLC, Chicago, IL, USA, version 7.1.2).

### 2.4. Evolutionary Analysis and Venn Diagram Construction

The full-length genomic sequences of representative begomoviruses and sequences isolated in this work were used to produce a phylogenetic tree using MEGA6.0. The alignment was performed using Muscle [44], and the function “Find Best DNA/Protein Models (ML)” was used to select the model for tree construction. The Bootstrap test (1000 replications) and the maximum likelihood (ML) method were employed for the begomovirus tree construction, along with the GTR + G + I model, with an alignment length of 2994 bp. Additionally, the full-length sequences of the alphasatellites were used for phylogenetic tree generation, aligned through Muscle, and the phylogenetic tree was constructed using with a Bootstrap test (1000 replications), ML method, and the T92 + G model, with an alignment length of 2043 bp. Gaps and missing data were eliminated during the analysis. For begomovirus, the outgroup sequence used was OW *Begomovirus caricachinaense* (papaya leaf curl China virus, PaLCuCNV). Accession numbers of sequences used for begomovirus phylogenetic tree construction are summarized in Table S2. For the alphasatellites, the betasatellite tomato leaf curl betasatellite (ToLCB) was used as an outgroup. Accession numbers of sequences used for alphasatellites phylogenetic tree construction are summarized in Table S3. In both trees, branches with partitions that appeared in less than 50% bootstrap tests were collapsed.

For the analysis of regulatory elements in begomovirus and alphasatellites, elements involved in replication and transcription were visualized in SnapGene software (GLS Biotech, LLC, Chicago, IL, USA, version 7.1.2) and identified by visual inspection according to previous reports [6,45,46,47].

In order to determine the overall distribution of the five DNA viral species identified by the CIDER-seq approach, specific primers for ToGMoV, ToSLCV, ToChLPV, WfaGA1, and WfaGA2 species were generated and used for end point PCR on the 21 surveyed tomato samples (Table S1). For Venn diagram construction, the results of PCR-based begomovirus and alphasatellite-specific detection from the completely surveyed samples (Figure S2) were counted for each component of the geminivirus complex (ToGMoV, ToSLCV, ToChLPV, WfaGA1, and WfaGA2), and the dataset was utilized to create a Venn diagram with the ggVennDiagram library in RStudio (2025.05.1 + 513).

### 2.5. Begomovirus–Alphasatellite Infectious Clones Agroinoculation and Viral Genomes Quantification

For the inoculation of begomovirus- and alphasatellite-derived infectious clones, *A. tumefaciens* strains harboring partial dimeric constructs were grown and inoculated as previously described [48]. *Solanum lycopersicum* Florida Lanai variety plants [49], at the four-leaf stage, were agroinoculated by stem puncture and leaf infiltration and maintained for four days in a BINDER bioclimatic chamber at 24 ºC under long-day conditions (16/8 light/darkness), 70% humidity, and then transferred to a greenhouse. The conditions in the latter corresponded to approximately 30 °C, 70% humidity, separation of 10 cm between plant pots, a photoperiod of approximately 14/10 light/darkness, and preventive treatment with Coragen^®^ pesticide (Syngenta, Stanton, CA, USA) every two weeks. Symptoms were recorded weekly, and samples of non-inoculated emerging apical leaves were collected at 14 and 28 dpi. For all experiments, three biological replicates with 8–12 plants per experimental condition were performed.

Viral genome quantification was determined by absolute quantitative PCR (qPCR). For qPCR assays, a CFX Opus Real-Time PCR System (Bio-Rad) was used. Each qPCR reaction contained 5 µL of Eva Green Supermix (Bio-Rad), 1 µL of each specific primer (10 µM), 1 µL of nuclease-free water, and 2 µL of DNA (200 ng) as a template. PCR plates containing the testing samples were subjected to one cycle at 95 °C for 2 min, followed by forty cycles at 95 °C for 5 s and 58 °C for 5 s. Melting curves were obtained after a final step of 60 cycles under a ramp rate of 0.5 °C every 5 s from 65 to 95 °C.

To create standard curves for absolute quantification, serial dilutions of viral genome monomeric plasmids containing between 1 × 10^7^ and 1 × 10^3^ molecules per µL were used. Standard curves were obtained by linear regression analysis of quantification cycle (Cq) values over the log_10_ of DNA concentration. Quantification of viral genomes was obtained by extrapolation of Cq data with the corresponding standard curve. To normalize qPCR data, the housekeeping EF1-α-derived amplicon was amplified from each sample. The resulting Cq values were used to calculate the differences between samples according to the 2^−Δct^ formula. Finally, absolute viral quantification was plotted as the number of viral genomes per microgram of total DNA on a log_10_ scale.

Statistical significance of the mean in each of the different agroinfection treatments at 14 dpi and 28 dpi was tested using the Fligner–Killeen test of homogeneity of variances, which was used to test the variance of the qPCR values. Then, the Wilcoxon–Mann–Whitney test was implemented to compare the means of the bioassay for single infections against triple infections of the three geminiviruses (ToGMoV, ToSLCV, ToChLPV) in their corresponding sections at 14 and 28 dpi. This was done to address the inequity between the number of samples across the different conditions that were compared in pairs, in addition to the fact that the qPCR values were all from the three different bioassays. No corrections for the pairwise comparisons were performed.

The same statistical procedure was used to compare the means of the qPCR values for the single infection of each begomovirus against the co-infection with WfaGA1, WfaGA2, and WfaGA1 + WfaGA2, separately by time of collection (14 and 28 dpi). All calculations were made in RStudio^®^ version 2025.05.1 + 513 with R version 4.3.3.

## 3. Results

### 3.1. A Leaf Curl Disease of Tomato Was Observed in Morelos, Mexico

**Figure 1:**
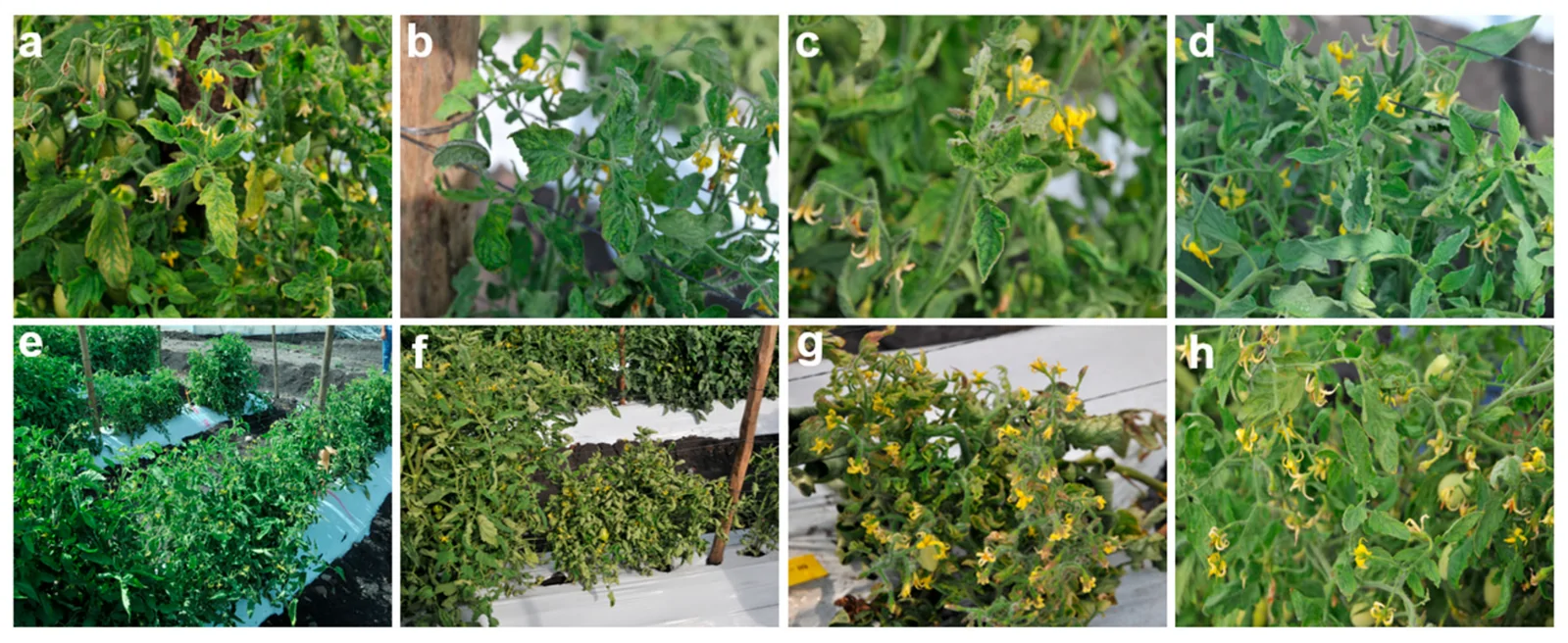
Survey of tomato plants showing begomovirus-related symptoms in Morelos, Mexico. Representative begomovirus-related symptoms were observed in tomato foliar and floral tissues. (**a**–**d**,**g**,**h**) Tomato plants showing leaf yellowing, interveinal chlorosis, leaf curling, and floral deformation. (**e**,**f**) Whole plants presenting dwarfism and general yellowing.

During 2018, tomato plants exhibiting symptoms associated with leaf curl disease of tomato, including leaf yellowing, interveinal chlorosis, leaf curling, dwarfism, and floral deformation (Figure 1), were observed in an open tomato field in Morelos, Mexico. Additionally, infestation by the *Bemisia tabaci* (whitefly) vector suggested a whitefly-associated viral disease etiology. A total of 21 symptomatic plants, including both leaf and fruit tissues, were collected and used for PCR-based molecular detection of begomoviruses using generalist primers. The results revealed the presence of begomoviruses in 20 of the 21 samples analyzed. Remarkably, only ~950 bp amplicons were detected (Figure S1), which, according to [32], putatively correspond to begomovirus bipartite species; however, their identity remains to be determined.

### 3.2. Circular DNA Enrichment Sequencing (CIDER-Seq) Revealed the Presence of a Novel Begomovirus–Alphasatellite Complex Associated with the Observed Leaf Curl Disease of Tomato

**Figure 2:**
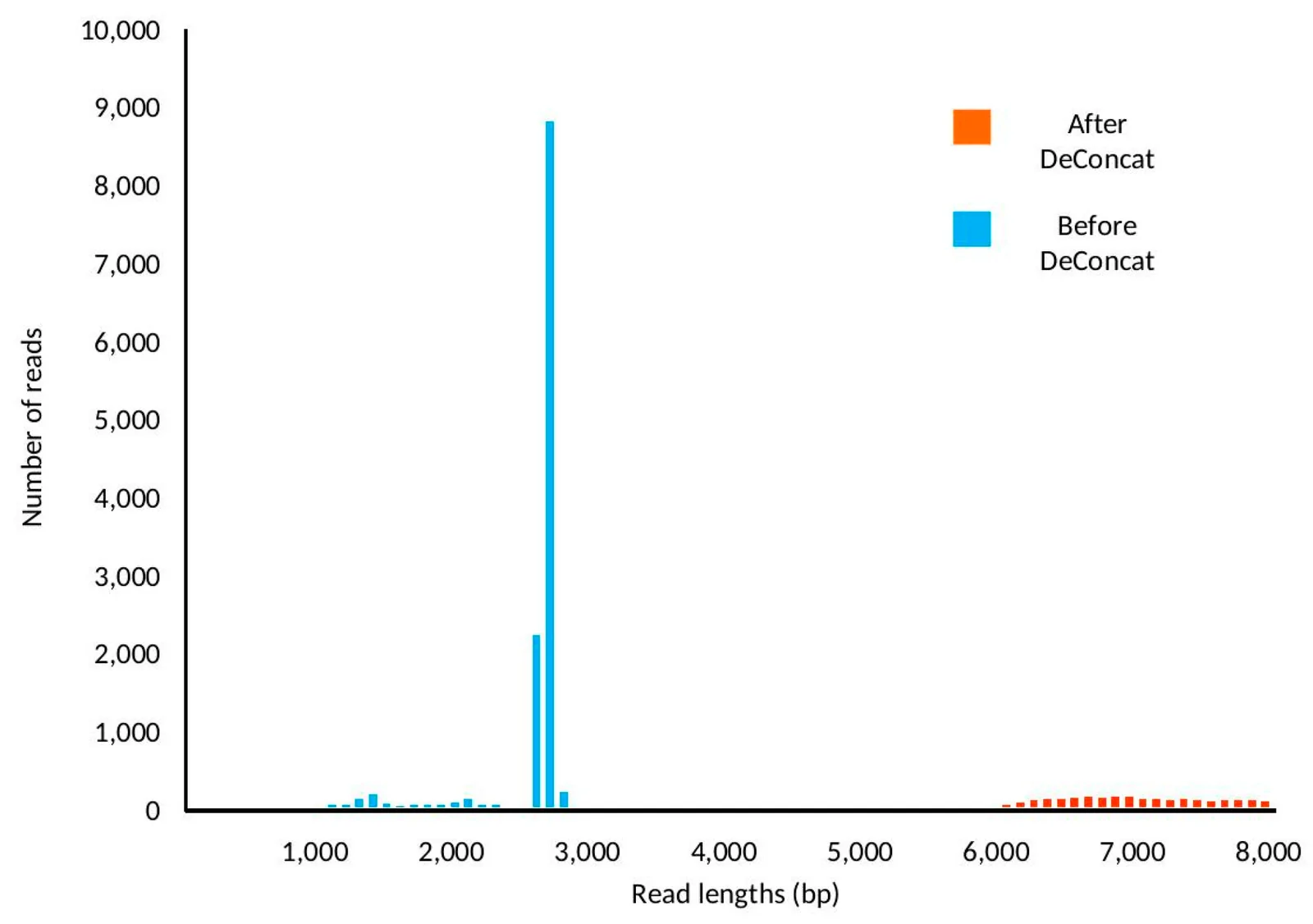
CIDER-Seq-derived long read sequencing of a Begomovirus-positive tomato sample. Bar plot presenting the frequency of the reads before (orange) and after (blue) deconcat processing.

A circular DNA enrichment sequencing (CIDER-Seq) method was employed to identify the begomovirus species associated with the observed leaf curl disease of tomato, utilizing a randomly selected begomovirus-positive sample (sample A20) for PacBio-derived library construction. The obtained sequence reads were demultiplexed utilizing SMRT Analysis software (Pacific Bioscience, Menlo Park, CA, USA) and processed with the CCS tool, achieving a >99% accuracy to generate high-fidelity (Hi-Fi) reads. A total of 16,326 Hi-Fi reads were produced, with a predominant population ranging from 6000 to 16,000 bp. Subsequently, CCS-processed Hi-Fi reads were deconcatenated utilizing the DeConcat algorithm, yielding 12,441 reads with a significantly diminished size distribution, exhibiting a distinct peak at ~2.7 kb, presumably corresponding to begomovirus genomes (Figure 2). The results corroborate the DeConcat algorithm procedure according to previous reports applied to begomovirus models [50]. Deconcatenated reads were mapped and annotated utilizing the *Solanum lycopersicum* genome sequence (GCF_000188115.5) as a reference, employing the programs minimap2 and BLAST+ tools. The unmapped sequences (putatively classified as viral genomes), ranging from 1000 to 3000 bp, were aligned beginning with the conserved motif “TAATATTAC” or “TAGTATTAC,” which contain the replication origin of ssDNA plant viral agents belonging to the *Geminiviridae* and *Nanoviridae/Alphasatellitidae* families, respectively.

Aligned sequences were annotated using the reference database of viral genomic sequences (ref_viruses_rep_genomes) through the BLAST+ tool, and by an in-house built R script using the “consensus” function from the seqinr library with the majority method. The alignments of each cognate group were used to generate single viral consensus sequences. The annotation of the consensus sequences led to the identification of three New World (NW) begomovirus species: tomato golden mottle virus (ToGMoV), comprising 6175 (49.63%) and 2513 (20.20%) of the total reads and exhibiting 97.09% and 97.30% identity with the respective DNA-A and DNA-B segments (Accession numbers: EF501976.1 and NC_008057.1, respectively); and tomato severe leaf curl virus (ToSLCV) and tomato chino La Paz virus (ToChLPV), which accounted for 3548 (28.52%) and 6 (0.05%) of the total reads, sharing 97.65% and 97.32% identity with the corresponding DNA-A segments (Accession numbers: DQ347947.1 and DQ347948.1), while their respective DNA-B segment were not identified (Table 1). Conversely, two begomovirus-associated alpha satellites previously identified in a whitefly vector-enabled metagenomic (VEM) survey in Guatemala [17], designated as whitefly-associated Guatemala alphasatellite 1 and 2 (WfaGA1 and WfaGA2) were identified. WfaGA1 accounted for 27 reads (0.22%), and WfaGA2 for 34 reads (0.27%), demonstrating 94.26% and 94.12% identity with their respective closest match sequences (Accession numbers: NC_038974.1 and NC_038973.1, respectively). Furthermore, 106 (0.85%) of the total reads were classified as putative circular molecules derived from the *Solanum lycopersicum* genome, while the remaining 32 (0.26%) reads were unclassified (Table 1). Notably, putative circular molecules originating from the *S. lycopersicum* genome varied in length from 60 to 9000 bp, and diverse cellular functions were detected, including RNA-associated molecules (tRNAs, snRNAs), transposons (LTRs, helitrons, LINEs), and pseudogenes.

### 3.3. Begomovirus–Alphasatellite Complex Genome Construction and CIDER-Seq Data Validation

**Figure 3:**
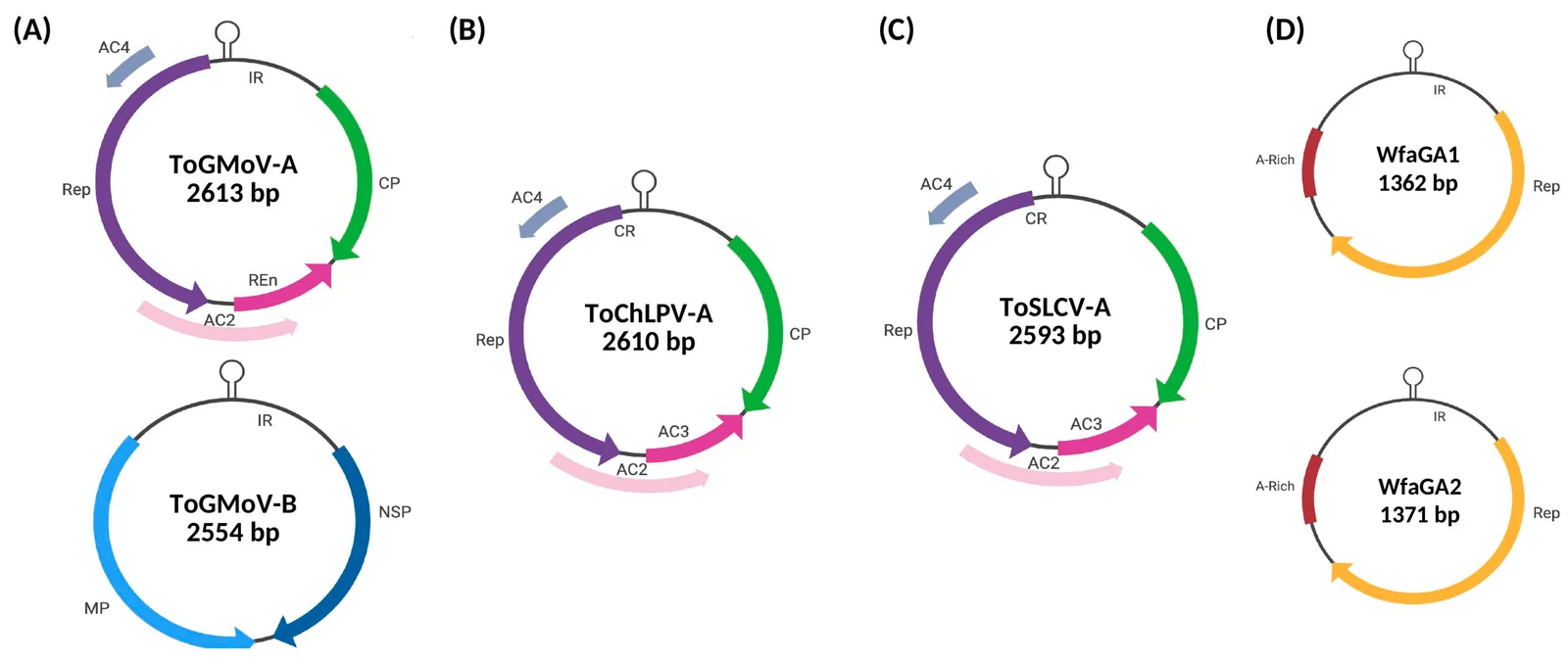
Begomovirus–alphasatellite complex full-length genomes. Begomovirus and alphasatellite full-length genomes were obtained through Sanger sequencing. For all viral genomes, relevant genomic features are indicated. (**A**) Bipartite, (**B**) and (**C**) Bipartite-like begomovirus genomes. ToGMoV, ToChLPV and ToSLCV DNA-A segments encode the CP gene in the virion sense, and the AC4, Rep, AC2 and AC3 genes in the complementary virion sense. The ToGMoV DNA-B segment encodes the NSP and MP genes in the virion sense and complementary virion sense, respectively. (**D**) Alphasatellite genomes. WfaGA1 and WfaGA2 encode a Rep-like gene in the virion sense and an adenine-rich (A-rich) zone. For all viral genomes, the intergenic (IR) region and replication origin are indicated by a continuous line and stem-loop structure, respectively.

Circular genomic maps were produced from the acquired viral consensus sequences. Among the begomovirus species, the bipartite genome of ToGMoV consists of 2614 nt for DNA-A and 2556 nt for DNA-B, while the bipartite-like (defined here as begomoviruses whose DNA-A has a genomic structure resembling that of bipartite New World begomoviruses in the absence of a cognate DNA-B) genomes of ToChLPV and ToSLCV comprise 2610 nt and 2593 nt for their respective DNA-A segments (Figure 3A, Figure 3B, and Figure 3C, respectively). For the three begomovirus species, the DNA-A segments showed the typical genomic arrangement of NW begomoviruses. In the virion-sense strand, the AV1/CP gene encodes the capsid protein. Moreover, four additional genes in the complementary sense are AC1/Rep, AC2/TrAP, AC3/REn, and AC4 [51]. For ToGMoV DNA-B, the BV1/NSP gene in the virion-sense strand and the BC1/MP gene in the complementary strands, involved in cell-to-cell and systemic viral movement, respectively, were predicted (Figure 3A–C). Conversely, circular genomes of 1362 nt and 1371 nt were acquired for the two alphasatellites WfaGA1 and WfaGA2, respectively. In both instances, a Rep-like gene and an adenine-rich region were identified in the virion-sense strand (Figure 3D). Both begomovirus and alphasatellite genomes possess a non-coding IR that contains the nonanucleotide TAATATTAC or TAGTATTAC, conserved in *Geminiviridae* and *Nanoviridae/Geminialphasatellitinae* families/sub-families, respectively [9]. Subsequently, to verify the accuracy of PacBio-derived consensus sequences of viral genomes, the complete complex comprising begomovirus and alphasatellite molecules was recovered through RCA or tail-to-tail PCR amplification, followed by cloning and sequencing of the full-length viral genomes. For each viral segment, two independent clones were fully sequenced using the Sanger method and aligned with the corresponding consensus sequence derived from PacBio. The findings indicate that the identity of the corresponding PacBio and Sanger sequences for both begomovirus and alphasatellite molecules is >99.9%. The obtained sequences were submitted to GenBank (Accession numbers: PZ400869, PZ400870, PZ400871, PZ400872, PZ400873, and PZ400874 for ToGMoV-A, ToGMoV-B, ToSLCV-A, ToChLPV-A, WfaGA2, and WfaGA1, respectively). Furthermore, to corroborate the absence of DNA-B segments associated with ToSLCV and ToChLPV, a PCR molecular detection using generalist primers directed at the DNA-B segment of *Begomovirus* genus members was used. Sample A20 was utilized for the CIDER-Seq approach, and supplementary random samples A12, A16, and A19 were tested. All tested samples exhibited the expected amplicon of 1128 bp. Following amplicon cloning and sequencing of all samples, an identity of >97% with ToGMoV DNA-B (Accession number: DQ406674) was observed (Figure S2). This data is in agreement with previous reports where cognate DNA-B associated with these begomovirus species has not been detected [30,52], suggesting their monopartite nature, which nevertheless remains to be biologically tested.

Altogether, these results support that long-read sequencing of circular viral genomes generated by the PacBio platform is comparable to that produced by the Sanger method, according to previous reports [53].

### 3.4. Begomovirus–Alphasatellite Complex Dynamics in Surveyed Plants Exhibiting Leaf Curl Disease of Tomato

**Figure 4:**
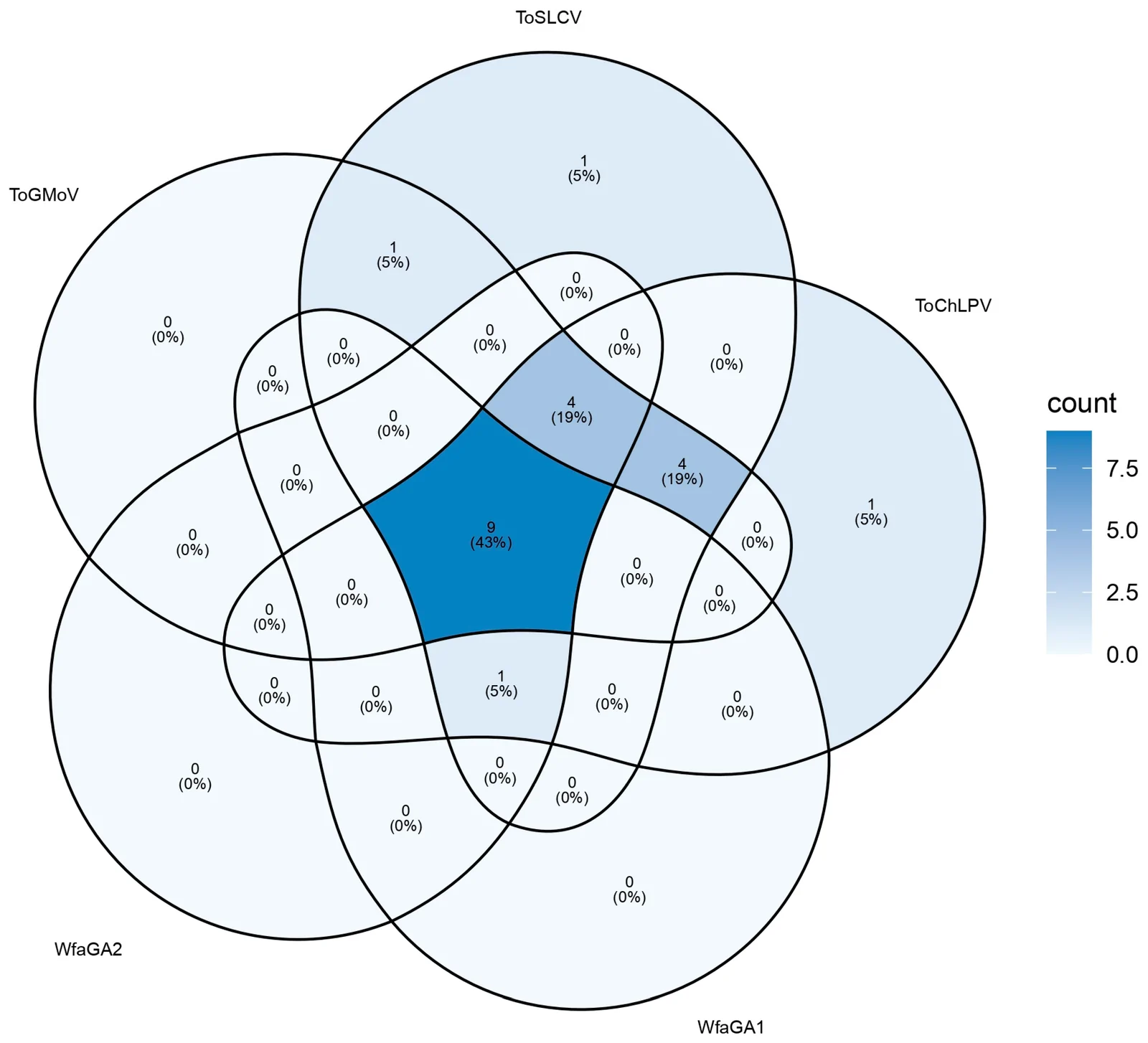
Venn diagram of begomovirus–alphasatellite complex dynamics in surveyed plants exhibiting leaf curl disease of tomato. For Venn diagram construction, the results of PCR-based begomovirus and alphasatellite-specific detection were counted for each component of the geminivirus complex (ToGMoV, ToSLCV, ToChLPV, WfaGA1, and WfaGA2), and the dataset was utilized to create a Venn diagram with the ggVennDiagram library in RStudio. The heat map indicated the frequency of different DNA viral molecule combinations.

In order to determine the prevalence of the different components of the begomovirus–aphasatellite complex in field conditions in plants exhibiting leaf curl disease of tomato, a PCR-based specific detection of each DNA viral component was performed for all 21 surveyed tomato samples (Figure S3). The PCR results were summarized and represented in a Venn diagram (Figure 4). Among the 21 analyzed tomato samples, the most prevalent infection configuration was the complex containing all five DNA viral and satellite DNA components identified by CIDER-Seq in sample A20 (ToGMoV, ToSLCV, ToChLPV, WfaGA1, and WfaGA2), representing 9 out of 21 (43%) of the total plants. Triple infections containing ToGMoV, ToSLCV, and WfaGA2 occurred in 4 out of 21 plants (19%), as well as ToGMoV, ToSLCV, and ToChLPV in another 4 out of 21 plants (19%). Additionally, there were minor occurrences of quadruple infections (ToSLCV, ToChLPV, WfaGA1, and WfaGA2), double infections (ToGMoV and ToSLCV), and single infections of either ToSLCV or ToChLPV, each represented by 1 out of 21 (5%) plants (Figure 4). These results suggest that the observed begomovirus–alphasatellite complex is predominantly established in field conditions.

### 3.5. Begomoviruses–Alphasatellite Complex Phylogenetic and Molecular Analyses

**Figure 5:**
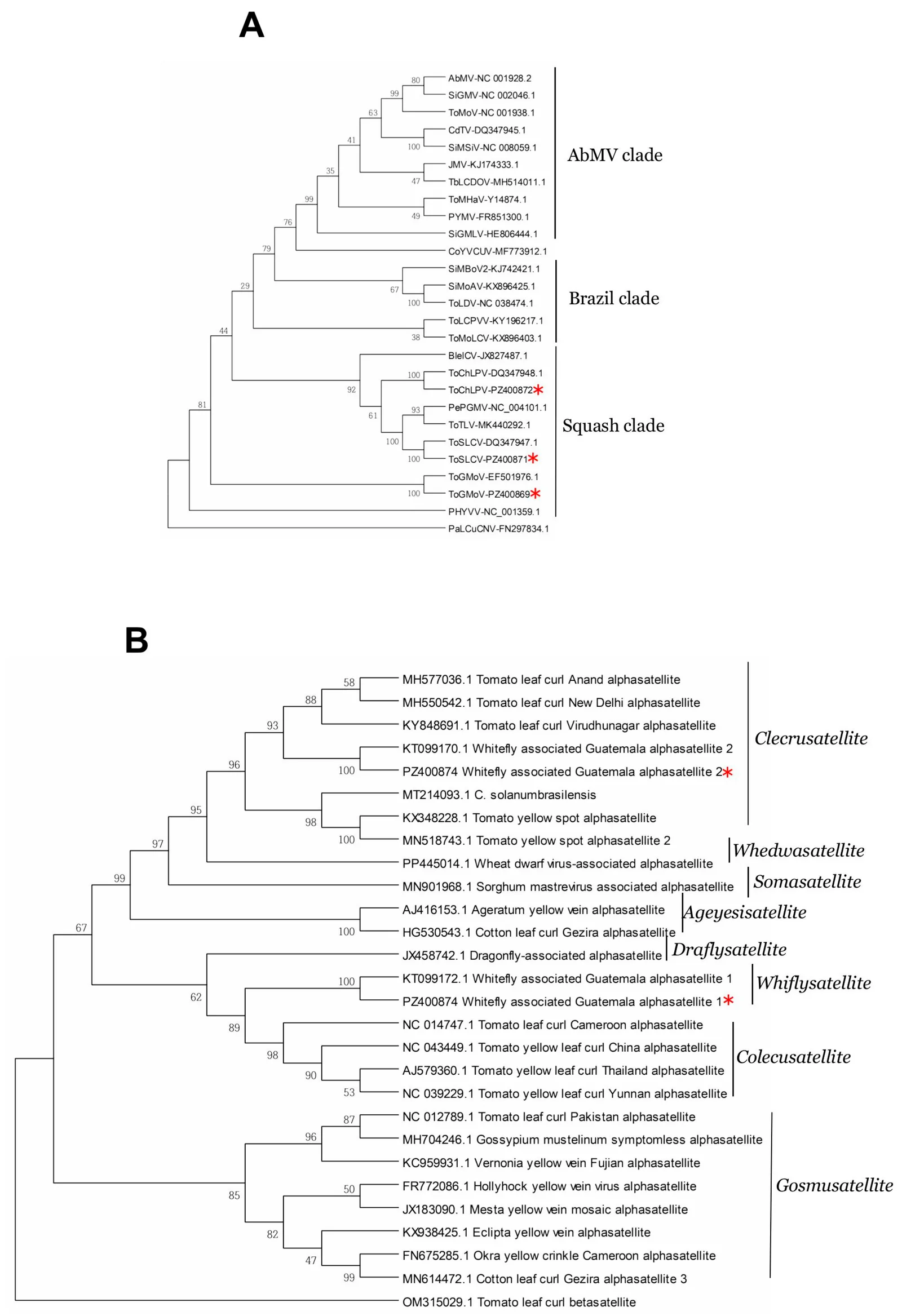
Phylogenetic trees of the begomovirus–alphasatellite complex. Phylogenetic trees were built using the ML method, GTR + G + I model for begomoviruses (**A**), T92 + G model for alphasatellites (**B**), and 1000 replicates of the Bootstrap test using the full-length genomic sequences obtained in this work. The numbers in the branches are the percentages of Bootstrap test that support the arrangement. DNA viral and satellite DNA components accession numbers are shown. Viral isolates obtained in the present study are indicated by red asterisks.

Phylogenetic and molecular analyses were conducted to predict the evolutionary relationship of the begomovirus–alphasatellite complex. The phylogenetic tree derived from the multiple alignment of complete nucleotide sequences of DNA-A segments from the begomovirus species identified in this study (ToGMoV, ToSLCV, and ToChLPV), alongside selected begomoviruses from the main clades present in the NW American continent (AbMV, Brazil, and Squash clade) available in the GenBank database, indicates that ToGMoV, ToSLCV, and ToChLPV are situated within the Squash clade, together with other NW begomovirus species adapted to pepper and tomato plants, such as PepGMV, PHYVV, and *Begomovirus solanumtortilis* (tomato twisted leaf virus; ToTLV) (Figure 5A).

Conversely, the phylogenetic tree was developed using the full-length genome sequences of representative members from eight genera of the *Geminialphasatellitinae* subfamily, grouping WfaGA1 and WfaGA2 in the *Whiflysatellite* and *Clecrusatellite* genera, respectively (Figure 5B). Interestingly, while various members of alphasatellites infecting tomato and classified within the *Clecrusatellite* genera have been reported in both OW and NW, WfaGA1 represents the sole member of the *Whiflysatellite* genera, identified through whitefly vector metagenomics [17]; its corresponding natural host remains unidentified.

### 3.6. Analysis of Regulatory Elements in the Begomovirus–Alphasatellite Complex

**Figure 6:**
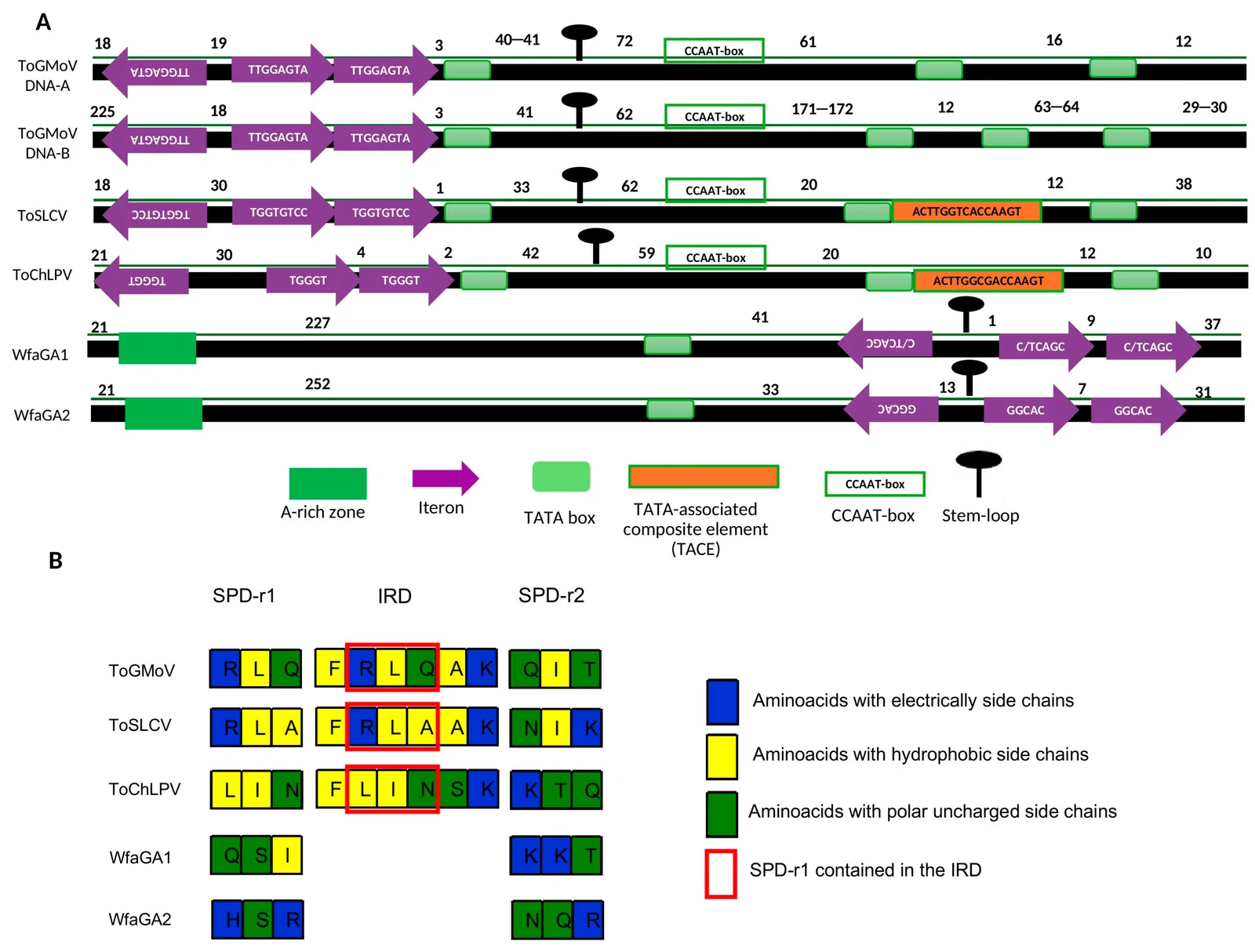
Regulatory elements in the begomovirus–alphasatellite complex. (**A**) Comparison of the IRs in the full-length genomes. The distance between the elements is presented as numbers displayed in bold. (**B**) Summary of the IRDs, SPD-r1s, and SPD-r2s in the complex. Square colors indicate the biochemical properties of amino acids and regions containing SDP elements.

An analysis of the regulatory elements associated with transcription and replication within the begomovirus-alphasatellite complex was performed. The begomovirus IR contains sequence motifs involved in viral gene expression and viral genome replication. The IR structure consists of a stem-loop arrangement with a conserved motif (TAATATTAC) flanked on the left side by the iterons and on the right side by the TATA boxes, which are essential for replication and transcription processes, respectively. The consensus sequence and arrangement of iterons facilitate the classification of geminivirus species based on their geophylogenetic (OW or NW) and host-adapted features [45]. Iterons represent specific union sites for the replication-associated (Rep) protein within the iteron-related domain (IRD), assisting in viral genome replication [46]. Moreover, additional specificity determinants (SPDs) clustered in two discrete Rep domains have been associated with high-affinity DNA binding activity [54]. The IRs of the three identified begomovirus species, ToGMoV, ToSLCV, and ToChLPV, displayed a typical NW iteron arrangement with two direct repeats and one inverted repeat located on the left side of the stem-loop structure and adjacent to a TATA box. The iterons of ToGMoV (DNA-A and DNA-B) contain a GGAG core and a TTGGAGTA consensus sequence, the iterons of ToSLCV have a GGTG core and a TGGTGTCC consensus sequence, and the iterons of ToChLPV have a GGTGT core and an ATGGGT consensus sequence (Figure 6A). Correspondingly, IRD and SPDs amino acid residues exhibited compatibility with their cognate iteron sequences according to previous reports [46]. IRDs containing FRLQ, FRLA, and FLIN amino acid sequences and SPDs (1 and 2) with RLQ/QSI, RLA/NIK, and QIT amino acid sequences were identified for ToGMoV, ToSLCV, and ToChLPV, respectively (Figure 6B). Interestingly, this iteron/IRD combination arrangement has also been documented in other NW tomato-infecting begomoviruses, such as *Begomovirus solanumaureimusivi* (tomato golden mosaic virus; TGMV), ToSLCV Guatemala isolate, and *Begomovirus solanumvariatuminvolutionis* (tomato mottle leaf curl virus; ToMoLCV).

Alphasatellites, or nanovirus-like satellites, possess a replication origin that displays all the sequence signatures typical of the *Nanoviridae* family and encode a single protein with significant similarity (45%) to the nanovirus master Rep protein [9]. Previous reports indicate that alphasatellite iterons are located on both sides of the stem-loop structure and contain the nanovirus-related nonanucleotide, TAGTATTAC, which is contiguous to a TATA box. Alphasatellite iterons demonstrate variability in copy number and positioning, with identified SPDs displaying some conservation among certain members; however, the chemical nature of these determinants is rather heterogeneous, including basic (R, K), acidic (E), strongly polar (Q), weakly polar (S, T) and non-polar (A, V, L) amino acid residues [54].The iterons of WfaGA1 and WfaGA2 alphasatellites displayed identical arrangements, featuring two direct and one inverted repeat relative to the stem-loop structure, but with distinct consensus sequences: C/TCAGC for WfaGA1 and GGCAC for WfaGA2, respectively. Additionally, SPD regions (SPD-r1 and SPD-r2) containing the residues QSI/KKT and HSR/NQR for WfaGA1 and WfaGA2, respectively, were identified (Figure 6A,B).

Despite the differences in both iterons and SPDs sequences between WfaGA1 and WfaGA2, a comparative analysis using representative members of *Clecrusatellite* genera from OW and NW revealed a tendency for conservation in SPDs-associated amino acid residues among those infecting tomato (Figure S4).

In OW and NW Clecrusatellites, including WfaGA2, the motif specificity determinant region 1 (SPD-r1) within their Rep-like protein amino acid sequence exhibited a predominant pattern comprising a neutral side chain amino acid residue (green), a positively charged side chain amino acid residue (blue), and another neutral side chain amino acid residue, with some exceptions where there was an amino acid residue with a non-polar side chain (yellow). The specificity determinant region 2 (SPD-r2) of Clecrusatellites exhibits greater conservation, characterized by the first two amino acid residues possessing uncharged side chains, and the third amino acid residue featuring a positively charged side chain. The Whiflysatellites, represented by a single member (WfaGA1), possess an SPD-r1 consisting of the initial two amino acid residues featuring uncharged side chains, followed by a final residue characterized by a nonpolar side chain. The difference in the biochemical composition of the WfaGA1 and WfaGA2 SPD motifs could result in variations in the interactions of Rep-like proteins with these motifs, due to the different ligand affinities.

### 3.7. ToSLCV and ToChLPV Are NW Monopartite Species, and ToGMoV Infection Showed a Host Recovery Phenotype

**Figure 7:**
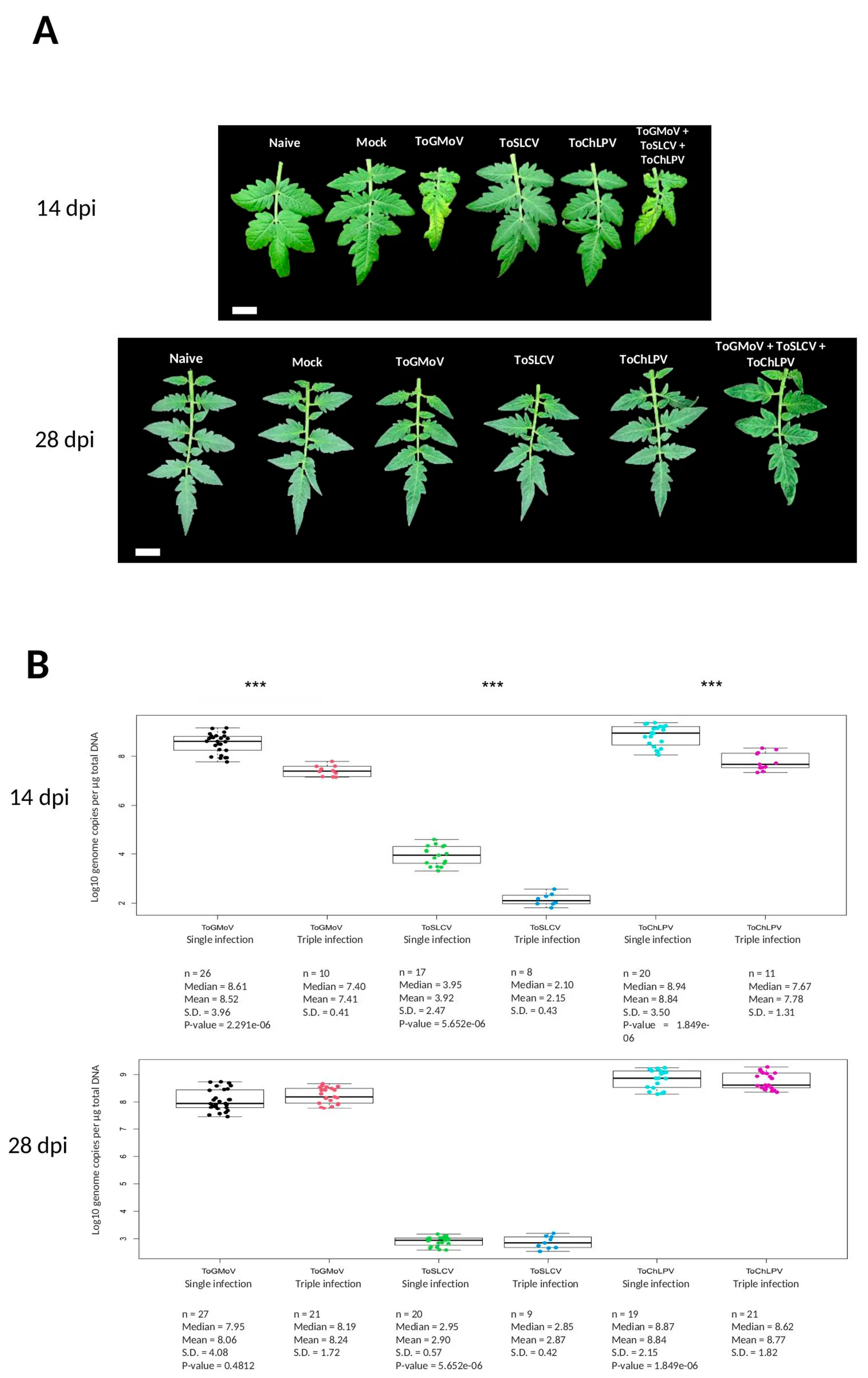
Bioassay to evaluate single and triple infections with ToGMoV, ToSLCV, and ToChLPV in begomoviruses. (**A**) Representative leaves from agroinfected plants at 14 dpi and 28 dpi. Scale bar = approximately 2 cm. (**B**) Means of the log10 genome copies per µg total DNA for the agroinfection conditions grouped by the time of sampling (14 dpi and 28 dpi). The means of the conditions were compared using the Wilcoxon–Mann–Whitney test. ***: *p* < 0.001. Each treatment includes the number of biological replicates (*n*), median, mean, standard deviation (s.d.), and *p*-value for each statistical comparison.

The CIDER-Seq approach previously identified three NW begomovirus species associated with the observed leaf curl disease of tomato: the bipartite species ToGMoV and two bipartite-like species, ToSLCV and ToChLPV, which lack the cognate DNA-B component. To determine the biological activity of these begomoviruses, infectious clones were constructed, and agroinfection-based assays were performed utilizing the *S. lycopersicum* Florida Lanai variety as a plant model. Fourteen days post-inoculation (14 dpi), tomato plants inoculated with ToGMoV exhibited typical begomovirus-related symptoms, including leaf curling and yellow mottling, while plants inoculated with ToSLCV or ToChLPV remained asymptomatic. The triple-inoculated ToGMoV/ToSLCV/ToChLPV plants exhibited stronger leaf curling and yellow mottling symptoms than the ToGMoV single-infected plants, suggesting a synergistic effect of mixed infection (Figure 7A, upper panel). Notably, after 28 dpi, viral symptoms disappeared completely in ToGMoV single-infected plants and were significantly reduced in triple infections (ToGMoV/ToSLCV/ToChLPV). Plants infected with ToSLCV and ToChLPV alone remained asymptomatic (Figure 7A, lower panel). Previously, host recovery phenotypes have been reported in other solanaceous plant-begomovirus interactions, such as the pepper-PepGMV interaction, a phenomenon associated with the host gene silencing antiviral mechanism [55]. Subsequently, viral genome levels were determined by absolute quantitative PCR (qPCR). The infection rate of the samples from the ToGMoV-infected plants was 100%, as they all showed the characteristic symptoms of ToGMoV during the three bioassays, calculated as the number positive/number inoculated. At 14 dpi in single infection, high levels of viral genomes were detected for ToGMoV (~1 × 10^8^ viral genomes, equivalent to 8 log_10_) and ToChLPV (~1 × 10^9^ viral genomes, equivalent to 9 log_10_), while ToSLCV exhibited lower levels (~1 × 10^4^ viral genomes, equivalent to 4 log_10_). It is noteworthy that in all cases of triple infection, the viral genomes exhibited a statistically significant decrease of approximately one order of magnitude, indicating a competitive effect in mixed infection (Figure 7B, upper panel). Finally, at 28 dpi, the viral genome levels for each begomovirus species were comparable in both single and triple infections. Viral genome levels of ~1 × 10^8^, corresponding to 8 log_10_, for ToGMoV; ~1 × 10^9^, equivalent to 9 log_10_ for ToChLPV; and ~1 × 10^3^, equivalent to 3 log_10_, for ToSLCV, were observed. It is important to highlight that in all independent biological assays, the infection efficiency was 100% for all begomovirus species tested according to qPCR detection. This, alongside the findings, demonstrates the monopartite nature of ToSLCV and ToChLPV, which can establish a systemic infection in the absence of a cognate DNA-B, corroborating earlier studies of NW monopartite begomovirus infecting tomato and non-cultivated plants in the Americas [21,56,57].

### 3.8. WfaGA1 and WfaGA2 Are Promiscuous Alphasatellites Associated with Multiple Begomovirus Helper Species

**Figure 8:**
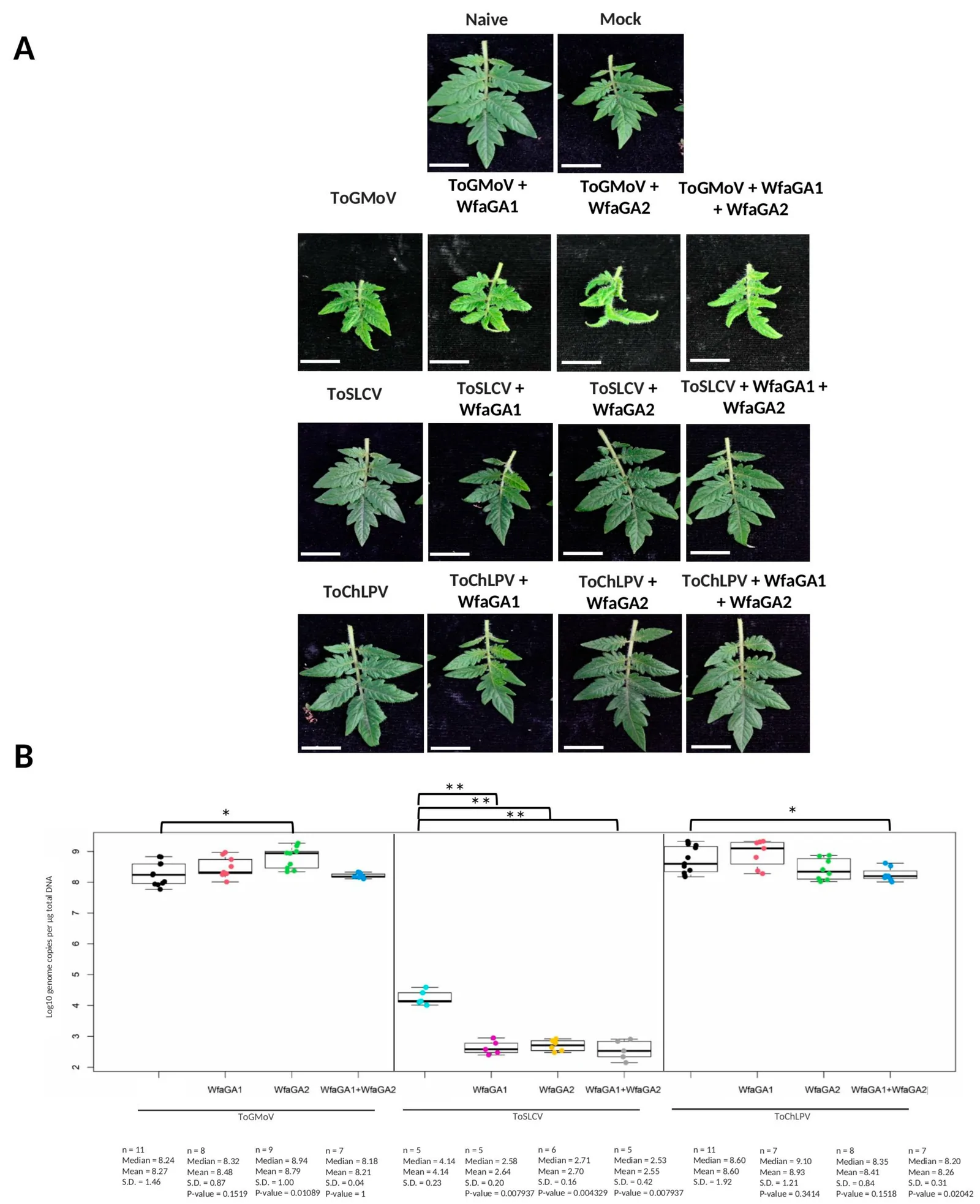
Bioassay to evaluate alphasatellite–begomovirus complex interaction. Co-inoculation of begomoviruses (ToGMoV, ToSLCV, or ToChLPV) and alphasatellites (WfaGA1 and WfaGA2) in single, double, and triple combinations was performed. (**A**) Representative leaves sampled from agroinfected plants at 14 dpi. (**B**) Means of the log10 genome copies per µg total DNA for the conditions of the agroinfections at 14 dpi were compared using the Wilcoxon–Mann–Whitney test in pairs with the single infection of each begomovirus. **: *p* < 0.01, *: *p* < 0.05. Each treatment includes the number of biological replicates (*n*), median, mean, standard deviation (s.d.), and *p*-value for each statistical comparison.

In addition to the three NW begomovirus species identified through the CIDER-Seq approach, two alphasatellite species were also associated with the observed leaf curl disease of tomato. To identify the helper virus species responsible for the systemic infection of WfaGA1 and WfaGA2, along with the biological implications of the interactions among multiple viral DNA molecules, begomovirus-alphasatellite co-inoculation assays utilizing agroinfectious clones were conducted in the *S. lycopersicum* Florida Lanai variety. At 14 dpi, the ToGMoV single-infected plants exhibited the previously documented symptoms of leaf curling and yellow mottling, while mixed infections involving ToGMoV and WfaGA1/WfaGA2 demonstrated severe symptoms. The intensity of symptoms was greatest in ToGMoV/WfaGA2, followed by ToGMoV/WfaGA1, and lastly ToGMoV/WfaGA1/WfaGA2 co-infections, which exhibited varying degrees of leaf curling, yellowing, and reduction in foliar area. Conversely, for ToSLCV and ToChLPV, all instances of single and double co-infections with WfaGA1 and WfaGA2 remained asymptomatic (Figure 8A). The begomovirus genomes were quantified using qPCR, and the infection rate of the three begomoviruses in all conditions showed positive signals (Figure 8B). Meanwhile, for the infections of ToSLCV, ToChLPV, and the alphasatellites at different rates, it was not possible to detect symptoms, but there was always a signal detected through qPCR for any of the other components in both single and mixed infections. A tendency for an increase in viral genomes was observed in ToGMoV/WfaGA1 co-infection, and a statistically significant increase in viral genomes of approximately one order of magnitude (~1 × 10^8^ viral genomes, equivalent to 8 log_10_, to ~1 × 10^9^ viral genomes, equivalent to 9 log_10_) was observed in the ToGMoV/WfaGA2 co-infection compared to the ToGMoV single infection. In contrast, no differences were detected in the triple infection ToGMoV/WfaGA1/WfaGA2, indicating a synergistic interaction effect of the alpahasatellite in the double co-infection and a competitive effect in the triple combination (Figure 8B). Conversely, an alphasatellite-driven antagonistic effect was noted for ToSLCV and ToChLPV. A statistically significant reduction in viral genomes of approximately one order of magnitude (~1 × 10^4^ viral genomes, equivalent to 4 log_10_, to ~1 × 10^3^ viral genomes, equivalent to 3 log_10_) was observed in all co-infections (ToSLCV/WfaGA1, ToSLCV/WfaGA2, and ToSLCV/WfaGA1/WfaGA2) compared to the ToSLCV single infection. Similarly, in the case of ToChLPV, a tendency toward viral genome reduction was observed in the ToChLPV/WfaGA2 co-infection, and a statistically significant reduction in viral genomes (~1 × 10^9^ viral genomes, equivalent to 9 log_10_, to ~1 × 10^8^ viral genomes, equivalent to 8 log_10_) was noted in the ToChLPV/WfaGA1/WfaGA2 co-infection (Figure 8B). At 28 dpi, all combinations of ToGMoV infection demonstrated host recovery, while the combinations of ToSLCV and ToChLPV with the alphasatellites remained asymptomatic. In all instances, no statistically significant differences in viral genomes were noted across all begomovirus–alphasatellite co-infections (Figure S5). Finally, to validate the alphasatellite systemic infection, both WfaGA1 and WfaGA2 were quantified in all co-inoculation assays. The results indicate that both alphasatellites induce systemic infections; however, their accumulation exhibited a stochastic distribution (Figure S6). Altogether, these findings demonstrate that the three begomovirus species (ToGMoV, ToSLCV, and ToChLPV) can act as helper viruses for both alphasatellites, and the presence of alphasatellite molecules during infections resulted in varied outcomes, including synergistic and antagonistic interactions.

## 4. Discussion

Viral diseases pose a significant threat to agriculture and result in devastating crop losses worldwide, with single-stranded DNA (ssDNA) viruses belonging to the *Geminiviridae* family, particularly those in the *Begomovirus* genus, representing the predominant group of emerging plant viruses [58]. Traditionally, virus research has focused on individual plant-virus interactions; however, it is now recognized that mixed infections of plant viruses are prevalent in nature, leading to significant diseases arising from interactions among causative agents [59]. Mixed infections of begomoviruses, including both inter- and intra-genus combinations, have been reported [60,61,62], and in certain cases, they are also found to be associated with circular ssDNA satellite molecules [6], leading to complex synergistic or antagonistic interactions.

Third-generation high-throughput sequencing (HTS) technologies based on long-read sequencing (i.e., PacBio and Oxford Nanopore platforms) have enabled the direct sequencing of full-length (>10 kb) molecules. An accurate and more extensive approach named the circular DNA enrichment sequencing (CIDER-Seq) method has been developed for DNA viral genomes, facilitating the identification of previously overlooked DNA viral entities [22].

This study reports for the first time a New World (NW) begomovirus–alphasatellite complex associated with leaf curl disease of tomato, identified using a CIDER-Seq approach in Morelos, Mexico. The obtained results expand the knowledge of DNA viral agents associated with the leaf curl disease of tomato in Mexico, highlighting the importance applying NGS tools in order to implement viromic-based epidemiological and ecological heuristic approaches.

The CIDER-Seq approach accurately revealed the presence of one bipartite begomovirus species, ToGMoV, two bipartite-like begomovirus species, ToSLCV and ToChLPV, and two begomovirus alphasatellies, WfaGA1, and WfaGA2. In spite of some complex components, including ToChLPV, WfaGA1 and WfaGA2, which were detected in low concentrations (0.05%, 0.22% and 0.027% of total reads), the PacBio versus Sanger comparative approach indicated that long-read sequencing of circular viral molecules generated by PacBio exhibited a concordance (>99%) with those produced by the Sanger method, thereby validating the accuracy of PacBio. Utilizing the CIDER-Seq technique, over 12,000 complete and highly accurate genomes of the African cassava mosaic virus (ACMV) begomovirus were acquired from RNAi-transgenic cassava plants in a field trial in Kenya, facilitating the identification of shifts in virus populations resulting from antiviral RNA interference pressure [22].

Interestingly, the putative cognate DNA B segments of the bipartite-like begomoviruses ToSLCV and ToChLPV were not identified in PacBio data or through PCR detection utilizing generalist primers directed to the begomovirus DNA-B segments. These data are in agreement with previous studies indicating unsuccessful detection of DNA-B in these two begomovirus species [30,52], suggesting their monopartite nature.

Additionally, a minor proportion of 0.85% of total reads were classified as putative circular molecules derived from the *Solanum lycopersicum* genome, associated with different cellular functions, including RNA-associated molecules (tRNAs, snRNAs), transposons (LTRs, helitrons, LINEs), and pseudogenes. Previous reports indicate that during stress exposure, plants release extrachromosomal circular DNA (eccDNA) from various genomic locations, which play crucial roles in plant adaptation to environmental challenges, including the amplification of glyphosate resistance in *Amaranthus palmeri* [63].

The dynamics of begomovirus–alphasatellite complex infection were determined in surveyed plants exhibiting leaf curl disease of tomato through viral-specific PCR molecular detection. The most predominant infection arrangement was the complex containing all five DNA viral species (ToGMoV, ToSLCV, ToChLPV, WfaGA1, and WfaGA2), indicating the possibility that WfaGA1 and WfaGA2 could associate promiscuously with different begomoviruses infecting tomato. For example, recently, through metagenomics, another tomato-infecting alphasatellite was reported in Brazil, *Clecrusatellite solanumbrsilensis* [16], leading to suspicion of a greater presence of alphasatellites that are part of begomovirus–alphasatellite complexes on the American continent, specifically in tomato crops.

Interestingly, both WfaGA1 and WfaGA2 were discovered through metagenomic analysis of whiteflies feeding on tomato plants in Guatemala [19], indicating their potential association with tomato-infecting helper begomoviruses. Whereas in the Americas, alphasatellites have been detected in association with begomoviruses infecting non-cultivated plants and crops, the biological characterization of begomovirus–alphasatellite mixed infections has been limited to experimental hosts (i.e., *N. benthamiana*) and testing artificial begomovirus–alphasatellite infections not found in field conditions [18]. The introduction of contaminated germplasm and viruliferous whitefly vectors containing begomovirus–alphasatellite complexes could be promoted by plant trade between Guatemala and Mexico, highlighting the importance of continuous epidemiological monitoring to prevent future outbreaks.

On the other hand, the phylogenetic tree derived from DNA-A segments of begomovirus species within the main clades of the New World (NW) (AbMV, Brazil, and Squash clade) indicated that ToGMoV, ToSLCV, and ToChLPV were grouped in the squash clade. The squash clade encompasses an atypical group of NW begomoviruses with more than 15 viral species distributed from Southern EUA to Brazil, initially causing devastating diseases in cucurbits (squash, melons, cucumbers), and subsequently causing severe infections in solanaceous crops (tomato, pepper, and eggplants) [34]. In Mexico, alongside ToGMoV, ToSLCV, and ToChLPV, pepper/tomato-infecting begomovirus species PHYVV have been associated with important diseases of tomato [32,52,64,65], suggesting that their phylogenetic proximity may be related to their ability to coexist within the begomovirus complex infecting solanaceous crops.

Conversely, the phylogenetic tree constructed using genome sequences from representative members of nine genera within the *Geminialphasatellitinae* subfamily clustered WfaGA1 and WfaGA2 into *Whiflysatellite* and *Clecrusatellite* genera, respectively. While alphasatellites from the *Clecrusatellie* genera infecting tomato in NW, including tomato yellow spot alphasatellite (ToYSA) in Argentina and Brazil [66] and *Clecrusatellite solanumbrasiliensis* in Brazil [16], have been previously documented, WfaGA1 is the sole representative of the *Whiflysatellite* genera.

Additionally, an analysis of regulatory elements located in the non-coding IR associated with transcription and replication within the begomovirus–alphasatellite complex was performed. The iteron organization of the three begomovirus species, ToGMoV, ToSLCV, and ToChLPV, exhibited a typical NW arrangement. Correspondingly, IRD and SPDs amino acid residues demonstrated compatibility with their cognate iteron sequences according to previous reports [46,54]. Furthermore, the IR regions of ToSLCV and ToChLPV exhibited a TATA-associated composite element (TACE) identified in NW begomoviruses of the Squash clade, which may play a role in TrAP-mediated derepression of the CP gene in vascular tissues [67]. The results suggest independent begomovirus genome replication, given that trans-replication would not be possible, based on several lines of experimental and indirect evidence showing a correlation between the Rep amino acid sequence and its predicted cognate DNA elements. Moreover, these iteron/IRD combination arrangements have been documented in other NW tomato-infecting begomoviruses [46].

Similarly, iterons and SPDs were determined for WfaGA1 and WfaGA2. The iterons of WfaGA1 and WfaGA2 alphasatellites exhibited an identical arrangement. Additionally, the SPD regions (SPD-r1 and SPD-r2) in a comparative analysis of representative members of *Clecrusatellite* genera from OW and NW revealed a tendency for conservation in SPDs-associated amino acid residues among those infecting tomato. The differential arrangement of WfaGA1 and WfaGA2 IR regulatory elements suggests different alphasatellite replication dynamics (i.e., Rep-iteron binding affinity, alphasatellite Rep-host replication machinery interaction, alphasatellite genome replication processivity). In the OW, including Pakistan, Southeast Asia, and China, alphasatellites have been associated with disease symptom amelioration, resulting in decreased accumulation of the helper viruses [8,11,12]. In the NW, certain begomovirus–alphasatellite complexes increase symptom severity and interfere with the transmission of the helper virus [18]. Therefore, biological characterization of NW begomovirus–alphasatellite complexes is essential to elucidate the ecological and epidemiological effects of these DNA viral agents on crops cultivated in the Americas.

To validate the biological activity of begomovirus-alphasatellite complexes, infectious clones were constructed, and agroinfection-based assays were developed using the *S. lycopersicum* Florida Lanai variety as a plant model. At 14 dpi, single infections of ToGMoV in begomovirus species exhibited characteristic symptoms, such as leaf curling and yellow mottling, while plants inoculated with ToSLCV and ToChLPV remained asymptomatic. It is now known that different plant virus species can cause asymptomatic (cryptic) infections; however, mixed infections have been reported to produce synergistic interactions with symptom-associated species or to act as beneficial mutualistic symbionts of the host plant [68,69]. The triple-inoculated ToGMoV/ToSLCV/ToChLPV plants showed stronger leaf curling and yellow mottling symptoms compared with ToGMoV single-infected plants, suggesting a synergistic mixed infection outcome. Remarkably, at 28 dpi, ToGMoV-related symptoms completely disappeared in single infections and were significantly reduced in ToGMoV/ToSLCV/ToChLPV triple infections. In some begomovirus–pepper (PHYVV or PepGMV) infections, a host recovery phenotype is observed after a symptomatic state. This host recovery phenotype has been associated with host defensive molecules and mechanisms, including salicylic acid, pathogen-related proteins (PRs), posttranscriptional gene silencing (PTGS), and transcriptional gene silencing (TGS) mediated by epigenetic defensive regulation [55,70,71]. However, during mixed PHYVV/PepGMV-pepper infection, host recovery is not observed or is partially abolished [60], in line with the observations made in our begomovirus complex. Complementary viral genome quantification showed that at 14 dpi, in single infections, whereas high genome levels of ToGMoV and ToChLPV were observed, lower genome levels were detected for ToSLCV. For triple infections, all three viral species showed a statistically significant decrease in viral genomes levels, and at 28 dpi, a similar tendency was observed.

These results demonstrate the monopartite nature of ToSLCV and ToChLPV, which can produce a systemic infection in tomato plants in the absence of a cognate DNA-B. It was believed that begomoviruses in NW were predominantly bipartite; however, various studies have described the existence of several NW begomoviruses infecting tomato plants with bipartite-like genomes but monopartite biological behavior, including *Begomovirus solanumdepravationis* (tomato leaf deformation virus; ToLDeV) in Ecuador and Peru [56], tomato mottle leaf curl virus (ToMoLCV), and *Begomovirus solanumviolavenae* (tomato leaf curl purple vein virus; ToLCPVV) in Brazil [72], and tomato twisted leaf virus (ToTLV) in Venezuela [73]. In bipartite begomoviruses, gene products encoded on the DNA-B are required for viral movement. Interestingly, it has been shown that monopartite begomoviruses belonging to the *Curtovirus* and *Topocuvirus* genera can complement bipartite movement functions, and in the case of the *Curtovirus betae* (beet curly top virus; BCTV), the V3 gene product and coat protein are implicated in viral movement [74]; future reverse genetic studies are necessary to determine the viral genes involved in the movement of this emerging group of NW monopartite begomoviruses.

Conversely, to identify the helper virus species assisting the systemic infection of WfaGA1 and WfaGA2, as well as the biological consequences of interactions among multiple viral DNA molecules, co-inoculation assays utilizing agroinfectious clones were performed. The results indicated that all three begomovirus species (ToGMoV, ToSLCV, and ToChLPV) can function as helper viruses; however, the interaction between ToGMoV and alphasatellites was synergistic, while the interactions between ToSLCV and ToChLPV and the satellite were antagonistic, affecting their genome replication capabilities, respectively. At 14 dpi, the symptom severity was higher in ToGMoV/WfaGA2, followed by ToGMoV/WfaGA1, and finally ToGMoV/WfaGA1/WfaGA2 co-infections, exhibiting different levels of leaf curling, yellowing, and foliar area reduction. In contrast, all single and double co-infections involving ToSLCV and ToChLPV with WfaGA1 and WfaGA2 remained asymptomatic. The differential interactions observed can be attributed to the relative contributions of two main factors: (1) competition between begomovirus and alphasatellite Rep proteins for cellular replication machinery, and (2) the suppression of gene silencing activity (PTGS and TGS) by alphasatellites [75], leading to different infection outcomes. Ultimately, to validate the systemic infection of the alphasatellites, both WfaGA1 and WfaGA2 were quantified in all co-inoculation assays, resulting in their positive detection in newly grown leaves and leading to the detection of systemic infections for both viral elements.

In the NW, alphasatellites have been detected so far in association with bipartite begomoviruses [13], but not monopartite ones, as shown in this study, and they are classified within the genus *Clecrusatellite*. However, the corresponding helper for the sole member of *Whiflysatellite*, WfaGA1, remained unidentified. Additionally, the different effects of WfaGA1 and WfaGA2 contrast with the tendency to associate a single effect of the alphasatellites with an associated begomovirus. But WfaGA2 has been shown to have the same capability as other clecrusatellites identified in Brazil, which were reported to increase the severity of symptoms. The symptoms caused by *Begomovirus euphorbiamusiviflavi* (euphorbia yellow mosaic virus; EuYMV) were more severe when co-infected with euphorbia yellow mosaic alphasatellite (EuYMA) in *N. benthamiana* and *Euphorbia heterophylla*, and these clecrusatellites appear to display a wide range of hosts and promiscuity in association with begomoviruses [18]. Since the detection of WfaGA1 and WfaGA2 by vector-enabled metagenomics (VEM) [19], this is the first report describing their associated begomovirus helper species, validating their biological activity.

In Mexico, the introduced OW begomovirus species TYLCV [25] and several NW begomovirus species, including CdTV [26], PepGMV [76], PHYVV [28], ToMoV [29], ToChLPV, ToSLCV, and ToGMoV [27,30,31,32], have been associated with leaf curl disease of tomato. ToGMoV and ToSLCV are begomovirus species introduced to the Mexican states of San Luis Potosi and Morelos from Central America (Honduras, Guatemala, and Nicaragua) [31,77], while ToChLPV appears to be endemic to Mexico [64]. Interestingly, both ToSLCV and ToChLPV are commonly found in co-infections in tomato and pepper crops alongside other begomoviruses such as TYLCV and PHYVV [52,78], indicating that these viral complexes are evolutionarily selected, with alphasatellites contributing additional complexity to the previously characterized begomovirus populations in Mexico. This is, to our knowledge, the first report of the presence of DNA satellites associated with begomoviruses in Mexico. Our data highlight the significance of global viral genomic surveillance utilizing NGS approaches for the continuous monitoring of plant viral pathogens, aimed at facilitating timely diagnostic and management strategies.

## 5. Conclusions

In this work, a novel begomovirus–alphasatellite complex has been identified with this kind of association for the first time in Mexico. It is composed of the begomoviruses ToGMoV, ToSLCV, and ToChLPV, along with the alphasatellites WfaGA1 and WfaGA2. The relationship of begomviruses with alphasatellites involves their role as helper viruses, albeit with different effects, making them a risk for tomato cultivation in Mexico. Globally, this activity is threatened by begomoviruses and occasionally in association with additional circular ssDNA satellite molecules, including beta, delta, and alphasatellites. In the Americas, the epidemiological and ecological role of alphasatellites and their helper begomovirus species is poorly understood. The CIDER-Seq approach has been established as an enrichment method that allows for a more complete scanning of the virome content and accurate long-read sequencing of viral-sized circular DNA molecules, including begomoviruses and their associated satellites. In Mexico, different begomovirus species are associated with the leaf curl disease of tomato, and by using CIDER-Seq, the role of alphasatellites in this disease is reported for the first time, challenging the development of effective strategies to minimize the damage caused by begomovirus complexes affecting tomato crops in the Americas.

## Figures and Tables

**Table 1:** Summary of data obtained from CIDER-Seq. Accession numbers of the closest relatives for each viral DNA species consensus sequence are shown.

	Deconcatenated Read Frequency (Total Number/Percentage)	Identity of Consensus Sequence’s Closest Relative (%/Accession Number)	Length of Circular Consensus Sequence (bp)
	3548/28.52%	ToSLCV (97.65%/DQ347947.1)	2593
	2513/20.20%	ToGMoV DNA-B (97.30%/NC_008057.1)	2556
	6175/49.63%	ToGMoV DNA-A (97.09%/EF501976.1)	2614
	34/0.27%	WfaGA2 (94.12%/NC_038973.1)	1371
	106/0.85%	*Solanum lycopersicum* ^a^ (NA)	NA
	27/0.22%	WfaGA1 (94.26%/NC_038974.1)	1362
	6/0.05%	ToChLPV (97.32%/DQ347948.1)	2610
	32/0.26%	Others ^b^	NA
**Total**	12,441/100		

^a^ For *S. lycopersicum*, different putative eccDNA molecules ranging from 60 to 9000 bp were identified. ^b^ For these reads, genomic annotation was not possible.

## Data Availability

The data sets generated for this study can be found in online repositories. The Sanger-derived sequences repository and accession numbers can be found here: “https://www.ncbi.nlm.nih.gov/genbank/ (accessed on 9 August 2026)”, PZ400869, PZ400870, PZ400871, PZ400872, PZ400873 and PZ400874. The raw data from the PacBio Library used in the present study is freely available at “https://www.ncbi.nlm.nih.gov/sra/PRJNA1465859 (accessed on 9 August 2026)”.

## Supplementary Materials

The following supporting information can be downloaded at https://www.mdpi.com/article/10.3390/v18090950/s1: Table S1: List of diagnostic and qPCR primers [79]; Table S2: Accession numbers of sequences used for begomovirus phylogenetic tree construction; Table S3: Accession numbers of sequences used for alphasatellite phylogenetic tree construction; Figure S1: Molecular detection of begomovirus in samples showing symptoms associated with leaf curl disease of tomato; Figure S2: Molecular detection of begomovirus-related DNA-B genomic segments in tomato samples from Morelos; Figure S3: Molecular detection of the begomovirus–alphasatellite complex in field samples showing symptoms associated with leaf curl disease of tomato; Figure S4: Regulatory elements of tomato-infecting alphasatellites belonging to the *Clecrusatellite* and *Whiflysatellite* genera in the Old World (OW) and New World (NW); Figure S5: Bioassay to evaluate the interaction between alphasatellites and begomoviruses; Figure S6: Quantification of alphasatellite titers in helper virus co-infections.

## References

[1] Fiallo-Olivé E., Lett J.-M., Martin D.P., Roumagnac P., Varsani A., Zerbini F.M., Navas-Castillo J. (2021). ICTV Virus Taxonomy Profile: Geminiviridae 2021. J. Gen. Virol..

[2] Rojas M.R., Macedo M.A., Maliano M.R., Soto-Aguilar M., Souza J.O., Briddon R.W., Kenyon L., Rivera Bustamante R.F., Zerbini F.M., Adkins S. (2018). World Management of Geminiviruses. Annu. Rev. Phytopathol..

[3] Rybicki E.P. (1994). A phylogenetic and evolutionary justification for three genera of Geminiviridae. Arch. Virol..

[4] Fondong V.N. (2013). Geminivirus protein structure and function. Mol. Plant Pathol..

[5] Orozco B.M., Hanley-Bowdoin L. (1996). A DNA structure is required for geminivirus replication origin function. J. Virol..

[6] Varma A., Singh M.K. (2024). The Role of Satellites in the Evolution of Begomoviruses. Viruses.

[7] Hassan I., Orílio A.F., Fiallo-Olivé E., Briddon R.W., Navas-Castillo J. (2016). Infectivity, effects on helper viruses and whitefly transmission of the deltasatellites associated with sweepoviruses (genus *Begomovirus*, family *Geminiviridae*). Sci. Rep..

[8] Nawaz-ul-Rehman M.S., Fauquet C.M. (2009). Evolution of geminiviruses and their satellites. FEBS Lett..

[9] Briddon R.W., Martin D.P., Roumagnac P., Navas-Castillo J., Fiallo-Olivé E., Moriones E., Lett J.M., Zerbini F.M., Varsani A. (2018). Alphasatellitidae: A new family with two subfamilies for the classification of geminivirus- and nanovirus-associated alphasatellites. Arch. Virol..

[10] Lefkowitz E.J., Dempsey D.M., Hendrickson R.C., Orton R.J., Siddell S.G., Smith D.B. (2018). Virus taxonomy: The database of the International Committee on Taxonomy of Viruses (ICTV). Nucleic Acids Res..

[11] Idris A.M., Shahid M.S., Briddon R.W., Khan A.J., Zhu J.K., Brown J.K. (2011). An unusual alphasatellite associated with monopartite begomoviruses attenuates symptoms and reduces betasatellite accumulation. J. Gen. Virol..

[12] Kumar M., Zarreen F., Chakraborty S. (2021). Roles of two distinct alphasatellites modulating geminivirus pathogenesis. Virol. J..

[13] Paprotka T., Metzler V., Jeske H. (2010). The first DNA 1-like α satellites in association with New World begomoviruses in natural infections. Virology.

[14] Jeske H., Kober S., Schäfer B., Strohmeier S. (2014). Circomics of Cuban geminiviruses reveals the first alpha-satellite DNA in the Caribbean. Virus Genes.

[15] Romay G., Chirinos D., Geraud-Pouey F., Desbiez C. (2010). Association of an atypical alphasatellite with a bipartite New World begomovirus. Arch. Virol..

[16] dos Reis L.d.N.A., Boiteux L.S., Fonseca M.E.N., Pereira-Carvalho R.d.C. (2025). Discovery of a novel alphasatellite of the genus Clecrusatellite associated with a wide array of New World tomato-infecting begomoviruses. Arch. Virol..

[17] Rosario K., Marr C., Varsani A., Kraberger S., Stainton D., Moriones E., Polston J., Breitbart M. (2016). Begomovirus-Associated Satellite DNA Diversity Captured Through Vector-Enabled Metagenomic (VEM) Surveys Using Whiteflies (Aleyrodidae). Viruses.

[18] Nogueira A.M., Nascimento M.B., Barbosa T.M.C., Quadros A.F.F., Gomes J.P.A., Orílio A.F., Barros D.R., Zerbini F.M. (2021). The association between new world alphasatellites and bipartite begomoviruses: Effects on infection and vector transmission. Pathogens.

[19] Rosario K., Seah Y., Marr C., Varsani A., Kraberger S., Stainton D., Moriones E., Polston J., Duffy S., Breitbart M. (2015). Vector-Enabled Metagenomic (VEM) Surveys Using Whiteflies (Aleyrodidae) Reveal Novel Begomovirus Species in the New and OldWorlds. Viruses.

[20] Idris A., Al-Saleh M., Piatek M., Al-Shahwan I., Ali S., Brown J. (2014). Viral Metagenomics: Analysis of Begomoviruses by Illumina High-Throughput Sequencing. Viruses.

[21] Guevara-Rivera E.A., Rodríguez-Negrete E.A., Aréchiga-Carvajal E.T., Leyva-López N.E., Méndez-Lozano J. (2022). From Metagenomics to Discovery of New Viral Species: Galium Leaf Distortion Virus, a Monopartite Begomovirus Endemic in Mexico. Front. Microbiol..

[22] Mehta D., Hirsch-Hoffmann M., Were M., Patrignani A., Zaidi S.S.E.A., Were H., Gruissem W., Vanderschuren H. (2019). A new full-length circular DNA sequencing method for viral-sized genomes reveals that RNAi transgenic plants provoke a shift in geminivirus populations in the field. Nucleic Acids Res..

[23] FAO (2009). El Estado Mundial de la Agricultura y la Alimentación. La Ganadería, a Examen.

[24] SIAP-SAGARPA Servicio De Información Agroalimentaria Y Pesquera, México. https://www.gob.mx/siap/acciones-y-programas/produccion-agricola-33119.

[25] Ascencio-Ibáñez J.T., Diaz-Plaza R., Méndez-Lozano J., Monsalve-Fonnegra Z.I., Argüello-Astorga G.R., Rivera-Bustamante R.F. (1999). First Report of Tomato Yellow Leaf Curl Geminivirus in Yucatán, México. Plant Dis..

[26] Idris A.M., Lee S.H., Brown J.K. (1999). First Report of Chino del Tomate and Pepper Hausteco Geminivurses in Greenhouse-Grown Tomato in Sonora, Mexico. Plant Dis..

[27] Holguín-Peña R.J., Vázquez Juárez R., Rivera-Bustamante R.F. (2003). First Report of a Geminivirus Associated with Leaf Curl in Baja California Peninsula Tomato Fields. Plant Dis..

[28] Garzón-Tiznado J.A., Torres-Pacheco I., Ascencio-Iba ez J.T., Herrera-Estrella L. (1993). Inoculation of Peppers with infectious Clones of a new Geminivirus by a Biolistic Procedure. Phytopathology.

[29] Garrido-Ramirez E.R., Gilbertson R.L. (1998). First Report of Tomato Mottle Geminivirus Infecting Tomatoes in Yucatan, Mexico. Plant Dis..

[30] Holguín-Peña R.J., Arguello-Astorga G.R., Brown J.K., Rivera-Bustamante R.F. (2006). A New Strain of Tomato chino La Paz virus Associated with a Leaf Curl Disease of Tomato in Baja California Sur, Mexico. Plant Dis..

[31] Mauricio-Castillo J.A., Argüello-Astorga G.R., Alpuche-Solís A.G., Monreal-Vargas C.T., Díaz-Gómez O., De La Torre-Almaraz R. (2006). First Report of Tomato severe leaf curl virus in México. Plant Dis..

[32] Mauricio-Castillo J.A., Argüello-Astorga G.R., Ambriz-Granados S., Alpuche-Solís A.G., Monreal-Vargas C.T. (2007). First Report of Tomato golden mottle virus on Lycopersicon esculentum and Solanum rostratum in Mexico. Plant Dis..

[33] Doyle, Doyle J. (1987). A rapid DNA isolation procedure for small quantities of fresh leaf tissue. Phytochem. Bull..

[34] Gregorio-Jorge J., Argüello-Astorga G.R., Frías-Treviño G., Bernal-Alcocer A., Moreno-Valenzuela O., Alpuche-Solís Á.G., Bañuelos-Hernández B., Hernández-Zepeda C. (2010). Analysis of a new strain of Euphorbia mosaic virus with distinct replication specificity unveils a lineage of begomoviruses with short Rep sequences in the DNA-B intergenic region. Virol. J..

[35] Inoue-Nagata A.K., Albuquerque L.C., Rocha W.B., Nagata T. (2004). A simple method for cloning the complete begomovirus genome using the bacteriophage 29 DNA polymerase. J. Virol. Methods.

[36] Hellens R.P., Anne Edwards E., Leyland N.R., Bean S., Mullineaux P.M. (2000). pGreen: A versatile and flexible binary Ti vector for Agrobacterium-mediated plant transformation. Plant Mol. Biol..

[37] Bang B., Lee J., Kim S., Park J., Nguyen T.T., Seo Y.S. (2014). A Rapid and Efficient Method for Construction of an Infectious Clone of Tomato yellow leaf curl virus. Plant Pathol. J..

[38] Mehta D., Cornet L., Hirsch-Hoffmann M., Zaidi S.S.-A., Vanderschuren H. (2020). Full-length sequencing of circular DNA viruses and extrachromosomal circular DNA using CIDER-Seq. Nat. Protoc..

[39] Li H. (2018). Minimap2: Pairwise alignment for nucleotide sequences. Bioinformatics.

[40] Danecek P., Bonfield J.K., Liddle J., Marshall J., Ohan V., Pollard M.O., Whitwham A., Keane T., McCarthy S.A., Davies R.M. (2021). Twelve years of SAMtools and BCFtools. Gigascience.

[41] García-Alcalde F., Okonechnikov K., Carbonell J., Cruz L.M., Götz S., Tarazona S., Dopazo J., Meyer T.F., Conesa A. (2012). Qualimap: Evaluating next-generation sequencing alignment data. Bioinformatics.

[42] Katoh K., Standley D.M. (2013). MAFFT Multiple Sequence Alignment Software Version 7: Improvements in Performance and Usability. Mol. Biol. Evol..

[43] Camacho C., Coulouris G., Avagyan V., Ma N., Papadopoulos J., Bealer K., Madden T.L. (2009). BLAST+: Architecture and applications. BMC Bioinform..

[44] Edgar R.C. (2004). MUSCLE: Multiple sequence alignment with high accuracy and high throughput. Nucleic Acids Res..

[45] Argüello-Astorga G.R., Guevara-González R.G., Herrera-Estrella L.R., Rivera-Bustamante R.F. (1994). Geminivirus Replication Origins Have a Group-Specific Organization of Iterative Elements: A Model for Replication. Virology.

[46] Argüello-Astorga G.R., Ruiz-Medrano R. (2001). An iteron-related domain is associated to Motif 1 in the replication proteins of geminiviruses: Identification of potential interacting amino acid-base pairs by a comparative approach. Arch. Virol..

[47] Mauricio-Castillo J.A., Torres-Herrera S.I., Cárdenas-Conejo Y., Pastor-Palacios G., Méndez-Lozano J., Argüello-Astorga G.R. (2014). A novel begomovirus isolated from sida contains putative cis- and trans-acting replication specificity determinants that have evolved independently in several geographical lineages. Arch. Virol..

[48] Cañizares M.C., Rosas-Díaz T., Rodríguez-Negrete E., Hogenhout S.A., Bedford I.D., Bejarano E.R., Navas-Castillo J., Moriones E. (2015). Arabidopsisthaliana, an experimental host for tomato yellow leaf curl disease-associated begomoviruses by agroinoculation and whitefly transmission. Plant Pathol..

[49] Rajabu C.A., Kennedy G.G., Ndunguru J., Ateka E.M., Tairo F., Hanley-Bowdoin L., Ascencio-Ibáñez J.T. (2018). Lanai: A small, fast growing tomato variety is an excellent model system for studying geminiviruses. J. Virol. Methods.

[50] Mehta D., Hirsch-Hoffmann M., Patrignani A., Gruissem W., Vanderschuren H. (2017). CIDER-Seq: Unbiased virus enrichment and single-read, full length genome sequencing. bioRxiv.

[51] Hanley-Bowdoin L., Bejarano E.R., Robertson D., Mansoor S. (2013). Geminiviruses: Masters at redirecting and reprogramming plant processes. Nat. Rev. Microbiol..

[52] Bañuelos-Hernández B., Mauricio-Castillo J.A., Cardenas-Conejo Y., Guevara-González R.G., Arguello-Astorga G.R. (2012). A new strain of tomato severe leaf curl virus and a unique variant of tomato yellow leaf curl virus from Mexico. Arch. Virol..

[53] Mehta D., Hirsch-hoffmann M., Were M., Patrignani A., Were H. (2018). A new full-length virus genome sequencing method reveals that antiviral RNAi changes geminivirus populations in field-grown cassava. bioRxiv.

[54] Londoño A., Riego-Ruiz L., Argüello-Astorga G.R. (2010). DNA-binding specificity determinants of replication proteins encoded by eukaryotic ssDNA viruses are adjacent to widely separated RCR conserved motifs. Arch. Virol..

[55] Rodríguez-Negrete E.A., Carrillo-Tripp J., Rivera-Bustamante R.F. (2009). RNA silencing against geminivirus: Complementary action of posttranscriptional gene silencing and transcriptional gene silencing in host recovery. J. Virol..

[56] Melgarejo T.A., Kon T., Rojas M.R., Paz-Carrasco L., Zerbini F.M., Gilbertson R.L. (2013). Characterization of a New World Monopartite Begomovirus Causing Leaf Curl Disease of Tomato in Ecuador and Peru Reveals a New Direction in Geminivirus Evolution. J. Virol..

[57] Zambrano K., Geraud-Pouey F., Chirinos D., Romay G., Marys E. (2011). Tomato chlorotic leaf distortion virus, a new bipartite begomovirus infecting Solanum lycopersicum and Capsicum chinense in Venezuela. Arch. Virol..

[58] García-Arenal F., Zerbini F.M. (2019). Life on the Edge: Geminiviruses at the Interface Between Crops and Wild Plant Hosts. Annu. Rev. Virol..

[59] Syller J. (2012). Facilitative and antagonistic interactions between plant viruses in mixed infections. Mol. Plant Pathol..

[60] Méndez-Lozano J., Torres-Pacheco I., Fauquet C.M., Rivera-Bustamante R.F. (2003). Interactions Between Geminiviruses in a Naturally Occurring Mixture: *Pepper huasteco virus* and *Pepper golden mosaic virus*. Phytopathology.

[61] Rentería-Canett I., Xoconostle-Cázares B., Ruiz-Medrano R., Rivera-Bustamante R.F. (2011). Geminivirus mixed infection on pepper plants: Synergistic interaction between PHYVV and PepGMV. Virol. J..

[62] Roberto Reveles Torres L., Velásquez Valle R., Armando Mauricio Castillo J., Torres R., Valle V., Castillo M., Salas Muñoz J.A. (2012). Detection of Mixed Infections Caused by Begomovirus and Curtovirus in Chili pepper for drying plants in San Luis Potosi, Mexico Detección de Infecciones Mixtas Causadas por Begomovirus y Curtovirus en Plantas de Chile para Secado en San Luis Potosí, Méxic. Rev. Mex. Fitopatol..

[63] Molin W.T., Yaguchi A., Blenner M., Saski C.A. (2020). The EccDNA replicon: A heritable, extranuclear vehicle that enables gene amplification and glyphosate resistance in amaranthus palmeri. Plant Cell.

[64] Holguín-Peña R.J., Vázquez-Juárez R., Rivera-Bustamante R.F. (2005). A New Begomovirus Causes Tomato Leaf Curl Disease in Baja California Sur, Mexico. Plant Dis..

[65] Moreno-Félix M.L., Rodríguez-Negrete E.A., Camacho-Beltrán E., Leyva-López N.E., Meléndrez-Bojórquez N., Méndez-Lozano J. (2018). A new isolate of Pepper huasteco yellow vein virus (PHYVV) breaks geminivirus tolerance in tomato (*Solanum lycopersicum*) commercial lines. Acta Hortic..

[66] Ferro C.G., Silva J.P., Xavier C.A.D., Godinho M.T., Lima A.T.M., Mar T.B., Lau D., Zerbini F.M. (2017). The ever increasing diversity of begomoviruses infecting non-cultivated hosts: New species from Sida spp. and Leonurus sibiricus, plus two New World alphasatellites. Ann. Appl. Biol..

[67] Cantú-Iris M., Pastor-Palacios G., Mauricio-Castillo J.A., Bañuelos-Hernández B., Avalos-Calleros J.A., Juárez-Reyes A., Rivera-Bustamante R., Argüello-Astorga G.R. (2019). Analysis of a new begomovirus unveils a composite element conserved in the CP gene promoters of several Geminiviridae genera: Clues to comprehend the complex regulation of late genes. PLoS ONE.

[68] Roossinck M.J. (2011). The good viruses: Viral mutualistic symbioses. Nat. Rev. Microbiol..

[69] Roossinck M.J., Bazán E.R. (2017). Symbiosis: Viruses as Intimate Partners. Annu. Rev. Virol..

[70] Carrillo-tripp J., Lozoya-Gloria E., Rivera-bustamante R.F. (2007). Symptom Remission and Specific Resistance of Pepper Plants After Infection by Pepper golden mosaic virus. Phytopatology.

[71] García-Neria M.A., Rivera-Bustamante R.F. (2011). Characterization of Geminivirus resistance in an accession of Capsicum chinense Jacq. Mol. Plant Microbe. Interact..

[72] Macedo M.A., Albuquerque L.C., Maliano M.R., Souza J.O., Rojas M.R., Inoue-Nagata A.K., Gilbertson R.L. (2018). Characterization of tomato leaf curl purple vein virus, a new monopartite New World begomovirus infecting tomato in Northeast Brazil. Arch. Virol..

[73] Romay G., Geraud-Pouey F., Chirinos D., Mahillon M., Gillis A., Mahillon J., Bragard C. (2019). Tomato Twisted Leaf Virus: A Novel Indigenous New World Monopartite Begomovirus Infecting Tomato in Venezuela. Viruses.

[74] Briddon R.W., Markham P.G., Centre J.I. (2001). Complementation of bipartite begomovirus movement functions by topocuviruses and curtoviruses Brief Report. Arch. Virol..

[75] Zhao L., Che X., Wang Z., Zhou X., Xie Y. (2022). Functional Characterization of Replication-Associated Proteins Encoded by Alphasatellites Identified in Yunnan Province, China. Viruses.

[76] Holguín-Peña R.J., Juárez R.V., Rivera-Bustamante R.F. (2004). Pepper golden mosaic virus Affecting Tomato Crops in the Baja California Peninsula, Mexico. Plant Dis..

[77] Rojas M.R., Hagen C., Lucas W.J., Gilbertson R.L. (2005). Exploiting Chinks in the Plant’s Armor: Evolution and Emergence of Geminiviruses. Annu. Rev. Phytopathol..

[78] Cardenas-Conejo Y., Argüello-Astorga G.R., Poghosyan A., Hernandez-Gonzalez J., Lebsky V., Holguin-Peña J., Medina-Hernandez D., Vega-Peña S. (2010). First Report of Tomato yellow leaf curl virus Co-infecting Pepper with Tomato chino La Paz virus in Baja California Sur, Mexico. Plant Dis..

[79] Rotenberg D., Thompson T.S., German T.L., Willis D.K. (2006). Methods for effective real-time RT-PCR analysis of virus-induced gene silencing. J. Virol. Methods.

