# Supplementary material for: Circular DNA Enrichment Sequencing (CIDER-Seq) Facilitated the Discovery of a New World (NW) Begomovirus–Alphasatellite Complex Associated with Leaf Curl Disease of Tomato in Morelos, Mexico
Source: Viruses. 2026 Aug 30;18(9):950. doi: 10.3390/v18090950 (PMC13611992; doi:10.3390/v18090950)
Supplement: Supplementary file 1 [file viruses-18-00950-s001.zip › viruses-4440557-supplementary.pdf]

Supplementary material

Supplementary Table S1. List of diagnostic and qPCR primers.

Primer name	Primer sequence (5´-3')	Size of the product (bp)	PCR usage	Reference
DRG-SAR	GAGTCTAGATGCTGACCTCCTCTAGCWGATCTGC	1100 (monopartite) /950 (bipartite)	Generalist detection of begomovirus DNA-A segment	[1]
CP-70	CACGGATCCGATTGRACCTTACANGGNCCTTCACA			
BV1-310-for	AGGWACRGTNAARATYGARCGTGT	1128	Generalist detection of begomovirus DNA-B segment	[2]
BC1- 290-rev	CCSATMAGRTGYAACATAGATCTCC			
ToGMoV-A-F	GTGAGCGAGCATAGTGGG	1209	Viral genome sequencing and specific detection	This study
ToGMoV-A-R	AAACCATATGTAAATCACG			
ToSLCV-F	GCAGGCTATAGTCAAGCG	999		
ToSLCV-R	CAATTATTTGAGTGGACACC			
ToChLPV-F	CCACACTAACAGCGTTATG	1262		
ToChLPV-R	ATTAGTATAATCATCGAGG			
ToAS-1-F	TTTGTCTCTGCCGGTGC	492		
ToAS-1-R	GTCACGAGTGGAAGCTGCG			
ToAS-2-F	AATTACGTTCTTCCTCTTTTTTCT	468		
ToAS-2-R	AGTTCGGTCATCAGGGC			
qPCR ToGMoV-F	GCAGTCTGCTGGCCTCC	177	Viral-specific quantification (qPCR)	
qPCR ToGMoV-R	GAACGAACGACAGTTCGACC			
qPCR ToSLCV-F	GTGAGTGAGGTCGAAAAATCTAGC	138		
qPCR ToSLCV-R	CCATGGTCAGTCGTCAAACC			
qPCR ToChLPV-F	GATACCAGGTCGAAGAATCGGC	144		
qPCR ToChLPV-R	ATTACGGTTGAGCACTCCGAC			
qPCR WfaGA1-F	ACCGTCGCCATTTGCAGG	188		
qPCR WfaGA1-R	ACAATAATCCCCAAATTCCCAGG			
qPCR WfaGA2-F	TGATGGAGCTCACATCGAACC	209		
qPCR WfaGA2R	TCGCCAGGCATCGACG			
EF1-F	GATTGGTGGAATTGGAACTGTC	130	qPCR Internal control	[3]
EF1-R	AGCTTCGTGGTGCATCTC			
ToChLPV genome-F	CCAATT**GAGCTC**TCTTCAAAAACTCTG	2628	Virus full-genome amplification (abutting primers)[a]	This study
ToChLPV genome-R	TGAAGA**GAGCTC**AATTGGGTATACC			
WfaGA1 genome-F	GCCCCT**GAGCTC**CTCC	1377		
WfaGA1 genome-R	GAG**GAGCT**CAGGGGCAC			
WfaGA2 genome-F	GCT**TCTAGA**TTTGCCGGGC	1383		
WfaGA2 genome-R	AAA**TCTAG**AAGCATCCTTTGTGC			

[a]Restriction sites SacI for ToChLPV and XbaI for WfaGA1-1, highlighted in bold, were used for the construction of hemidimeric infectious clones.

References

1. Mauricio-Castillo, J.A.; Argüello-Astorga, G.R.; Ambriz-Granados, S.; Alpuche-Solís, A.G.; Monreal-Vargas, C.T. First Report of Tomato golden mottle virus on Lycopersicon esculentum and Solanum rostratum in Mexico . *Plant Dis.* **2007**, *91*, 1513–1513.

2. Gregorio-Jorge, J.; Argüello-Astorga, G.R.; Frías-Treviño, G.; Bernal-Alcocer, A.; Moreno-Valenzuela, O.; Alpuche-Solís, Á.G.; Bañuelos-Hernández, B.; Hernández-Zepeda, C. Analysis of a new strain of Euphorbia mosaic virus with distinct replication specificity unveils a lineage of begomoviruses with short Rep sequences in the DNA-B intergenic

region. *Virol. J.* **2010**.

3. Rotenberg, D.; Thompson, T.S.; German, T.L.; Willis, D.K. Methods for effective real-time RT-PCR analysis of virus-induced gene silencing. *J. Virol. Methods* **2006**, *138*, 49–59.

Supplementary Table S2. Accession numbers of sequences used for begomovirus phylogenetic tree construction.

Begomovirus name	Acronym	Accession Number
Begomovirus bauri (Abutilon mosaic virus)	AbMV	NC_001928.2
Begomovirus sidaureidis (Sida golden mosaic virus)	SiGMV	NC_002046.1
Begomovirus solanumvariati (tomato mottle virus)	ToMoV	NC_001938.1
Begomovirus solanumflavi (chino del tomate virus)	CdTV	DQ347945.1
Begomovirus sidasinaloaense (Sida mosaic Sinaloa virus)	SiMSiV	NC_008059.1
Begomovirus jatrophamusivi (Jatropha mosaic virus)	JMV	KJ174333.1
Begomovirus nicotianadominicanense (tobacco leaf curl Dominican Republic virus)	TbLCDOV	MH514011.1
Begomovirus solanumhavanaense (tomato mosaic Havana virus)	ToMHaV	Y14874.1
Begomovirus tuberosi (potato yellow mosaic virus)	PYMV	FR851300.1
Sida golden mosaic Liguanea virus	SiGMLV	HE806444.1
Begomovirus corchocubaense (Corchorus yellow vein Cuba virus)	CoYVCUV	MF773912.1
Begomovirus sidaboliviaensecundi (Sida mosaic Bolivia virus 2)	SiMBoV2	KJ742421.1
Begomovirus sidavariatialagoense (Sida mottle Alagoas virus)	SiMoAV	KX896425.1
Begomovirus solanumcontorsionis (tomato leaf distortion virus)	ToLDV	NC_038474.1
Begomovirus solanumviolavenae (tomato leaf curl purple vein virus)	ToLCPVV	KY196217.1
Begomovirus solanumvariatuminvolutionis (tomato mottle leaf curl virus)	ToMoLCV	KX896403.1
Begomovirus solanumaureivariati (tomato golden mottle virus)	ToGMoV	EF501976.1
Begomovirus solanumaureivariati (tomato golden mottle virus)	ToGMoV	PZ400869
Begomovirus blechi (Blechum interveinal chlorosis virus)	BleICV	JX827487.1
Begomovirus solanumlapazense (tomato chino La Paz virus)	ToChLPV	DQ347948.1
Begomovirus solanumlapazense (tomato chino La Paz virus)	ToChLPV	PZ400872
Begomovirus capsicummusivi (pepper golden mosaic virus)	PePGMV	NC_004101.1
Begomovirus solanumtortilis (tomato twisted leaf virus)	ToTLV	MK440292.1
Begomovirus solanumseveri (tomato severe leaf curl virus)	ToSLCV	ToSLCV
Begomovirus solanumseveri (tomato severe leaf curl virus)	PZ400871	ToSLCV
Begomovirus capsicumhuastecoense (pepper huasteco yellow vein virus)	NC_001359.1	PHYVV
Begomovirus caricachinaense (papaya leaf curl China virus)	FN297834.1	PaLCuCNV

Supplementary Table S3. Accession numbers of sequences used for alphasatellite phylogenetic tree construction.

Alphasatellite name	Acronym	Accession Numbers
Tomato yellow spot alphasatellite	ToYSA	KX348228.1
Tomato yellow spot alphasatellite 2	ToYSA-2	MN518743.1
C. solanumbrasilensis	-	MT214093.1
Tomato leaf curl Anand alphasatellite	ToLCAA	MH577036.1
Tomato leaf curl Virudhunagar alphasatellite	ToLCVA	KY848691.1
Tomato leaf curl New Delhi alphasatellite	ToLCNDA	MH550542.1
Whitefly associated Guatemala alphasatellite 2	WfaGA2	KT099170.1
Whitefly associated Guatemala alphasatellite 2	WfaGA2	PZ400874
Sorghum mastrevirus associated alphasatellite	SMasA	MN901968.1
Ageratum yellow vein alphasatellite	AYVA	AJ416153.1
Cotton leaf curl Gezira alphasatellite	CLCuGA	HG530543.1
Tomato leaf curl Pakistan alphasatellite	ToLCPA	NC_012789.1
Gossypium mustelinum symptomless alphasatellite	GMSA	MH704246.1
Vernonia yellow vein Fujian alphasatellite	VeYVFjA	KC959931.1
Hollyhock yellow vein virus alphasatellite	HoYVA	FR772086.1
Mesta yellow vein mosaic alphasatellite	MEYVMA	JX183090.1
Eclipta yellow vein alphasatellite	EYVA	KX938425.1
Okra yellow crinkle Cameroon alphasatellite	OYCrCMA	FN675285.1
Cotton leaf curl Gezira alphasatellite 3	CLCuGA-3	MN614472.1
Dragonfly-associated alphasatellite	DfaA	JX458742.1
Whitefly associated Guatemala alphasatellite 1	WfaGA1	KT099172.1
Whitefly associated Guatemala alphasatellite 1	WfaGA1	PZ400874
Tomato leaf curl Cameroon alphasatellite	ToLCCA	NC_014747.1
Tomato yellow leaf curl Thailand alphasatellite	TYLCTHA	AJ579360.1
Tomato yellow leaf curl Yunnan alphasatellite	TYLCYnA	NC_039229.1
Tomato yellow leaf curl China alphasatellite	TYLCCNA	NC_043449.1
Wheat dwarf virus-associated alphasatellite	WDVaA	PP445014.1
Tomato leaf curl betasatellite	ToLCB	OM315029.1

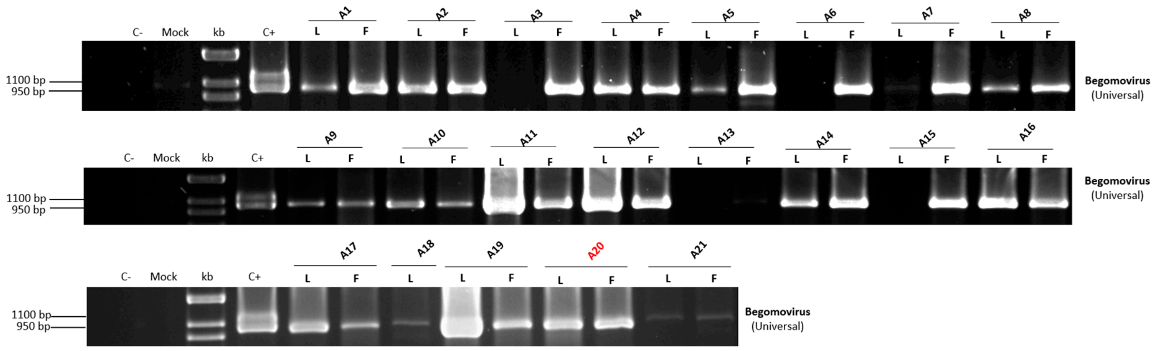

Supplementary Figure S1. Molecular detection of begomovirus in samples showing symptoms associated with leaf curl disease of tomato**.** Total DNA from leaf or fruit (L or F) tissue of 21 tomato plants (A1-A21) was used as the template for molecular detection by PCR using begomovirus generalist primers. C-, non-template negative control; Mock, healthy plant negative control; kb, molecular marker 1 kb (Thermo Scientific); C+, positive control samples infected with a mix of monopartite and bipartite begomoviruses. The expected amplicon molecular weights are indicated (1100 and 950 bp for monopartite and bipartite begomovirus, respectively). Sample A20, highlighted in red, was selected for PacBio sequencing.

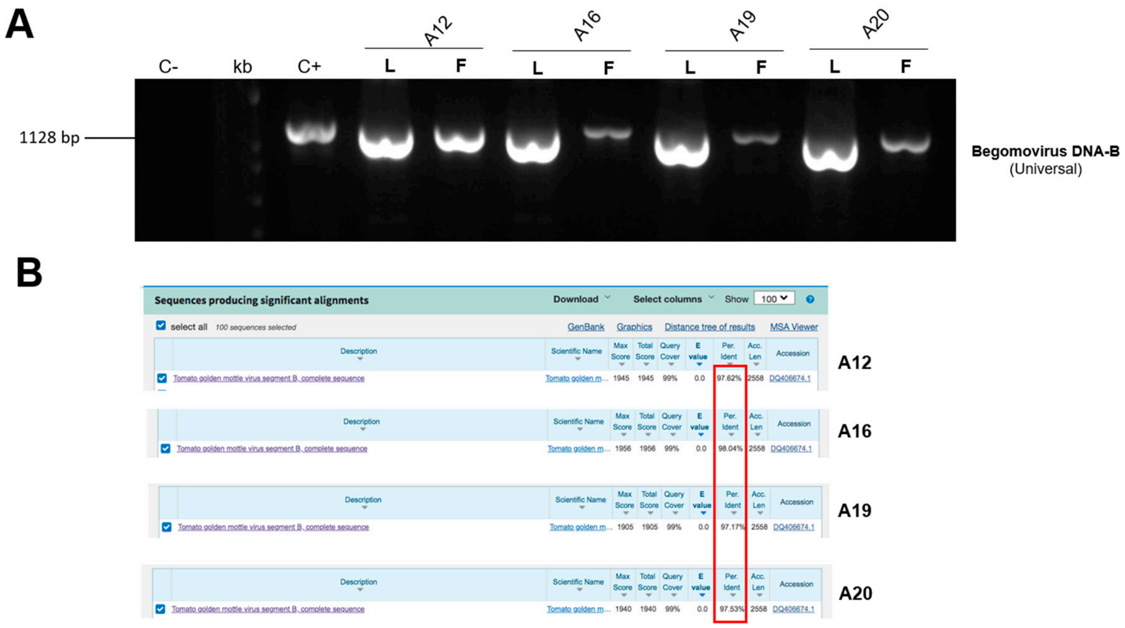

Supplementary Figure S2. Molecular detection of begomovirus-related DNA-B genomic segments in tomato samples from Morelos. (**a**) Total DNA from leaf or fruit (L or F) tissue of different tomato lines was used as a template for molecular detection using PCR. Tomato line codes are indicated. C-, non-template reaction negative control; Mock, healthy plant negative control; kb, molecular marker 1 kb (Thermo Scientific); C+, positive control of plasmidic DNA containing ToGMoV DNA-A or ToSLCV DNA-A (upper and lower panels, respectively). The molecular weight of the expected amplicons is indicated (1128 bp). Samples highlighted in red were selected for DNA-B detection using begomovirus DNA-B universal primers. (**b**) BLAST results from the core nucleotide database (core_nt) using the isolated PCR products of the samples are included for their identification: ToGMoV DNA-B (DQ406674.1).

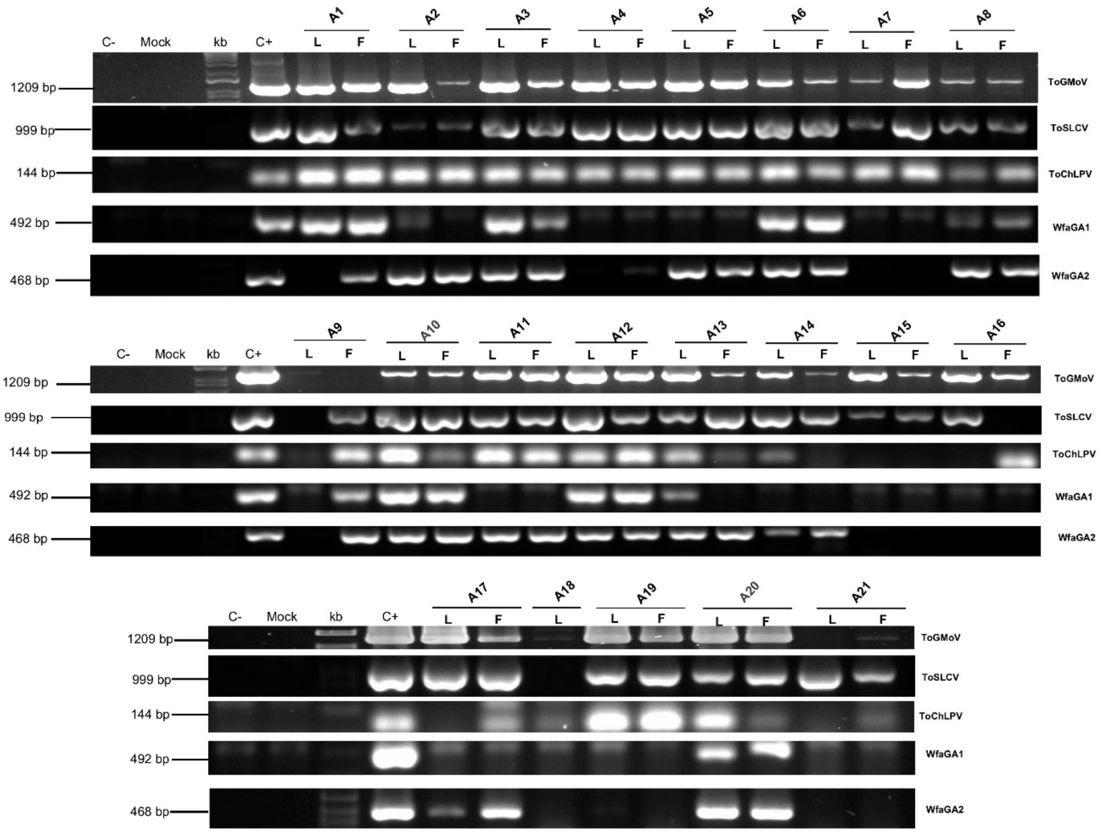

Supplementary Figure S3. Molecular detection of the begomovirus–alphasatellite complex in field samples showing symptoms associated with leaf curl disease of tomato. Using total DNA (100 ng) of 21 symptomatic and asymptomatic samples of *S. lycopersicum* from a tomato field of Morelos, PCRs were conducted to detect the presence of begomovirus using degenerate primers. The expected sizes of the resulting bands were 1100 bp (monopartite) and 950 bp (bipartite) (supplementary figure 1). Subsequently, specific molecular detections were carried out for ToGMoV, ToSLCV, ToChLPV, WfaGA1, and WfaGA2 in a second PCR. C-, negative non-DNA control; Mock, symptomless plant; kb, molecular weight marker; C+, vectors containing ToGMoV and ToSLCV isolates used as positive controls; L: leaf tissue; F: fruit tissue. The sizes of the amplicons were 999 bp (ToSLCV), 150 bp (ToChLPV), 1209 bp (ToGMoV), 492 bp (WfaGA1), and 468 bp (WfaGA2).

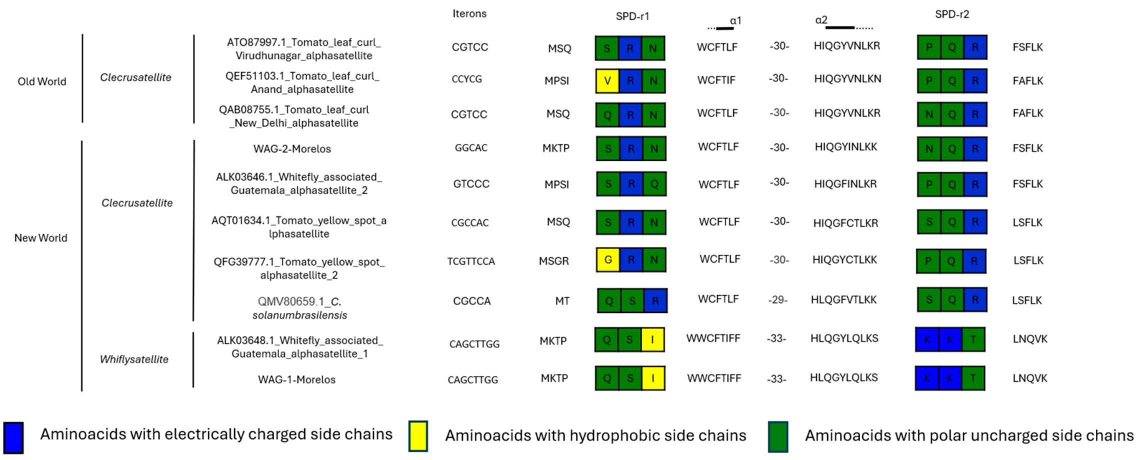

Supplementary Figure S4. Regulatory elements of tomato-infecting alphasatellites belonging to the *Clecrusatellite* and *Whiflysatellite* genera in the Old World (OW) and New World (NW). Representative amino acid sequences of Rep-like proteins from the alphasatellite genera *Clecrusatellite* and *Whiflysatellite* are displayed according to an Old World and New World division. The putative SPD sites 1 and 2 are encased in colored squares. The colors in the boxes represent the amino acid group to which each one belongs: blue (amino acids with electrically positively charged side chains), green (amino acids with side chains without charge), and yellow (amino acids with no polar side chains). The clusters are named SPD region 1 (SPD-r1) and SPD region 2 (SPD-r2). The motifs with conserved regions that constitute $\alpha1$ and $\alpha2$ are placed at the top of the alignment. The amino acid motifs and iteron sequences correspond to the sequences whose accession codes are placed on their left side.

A

B

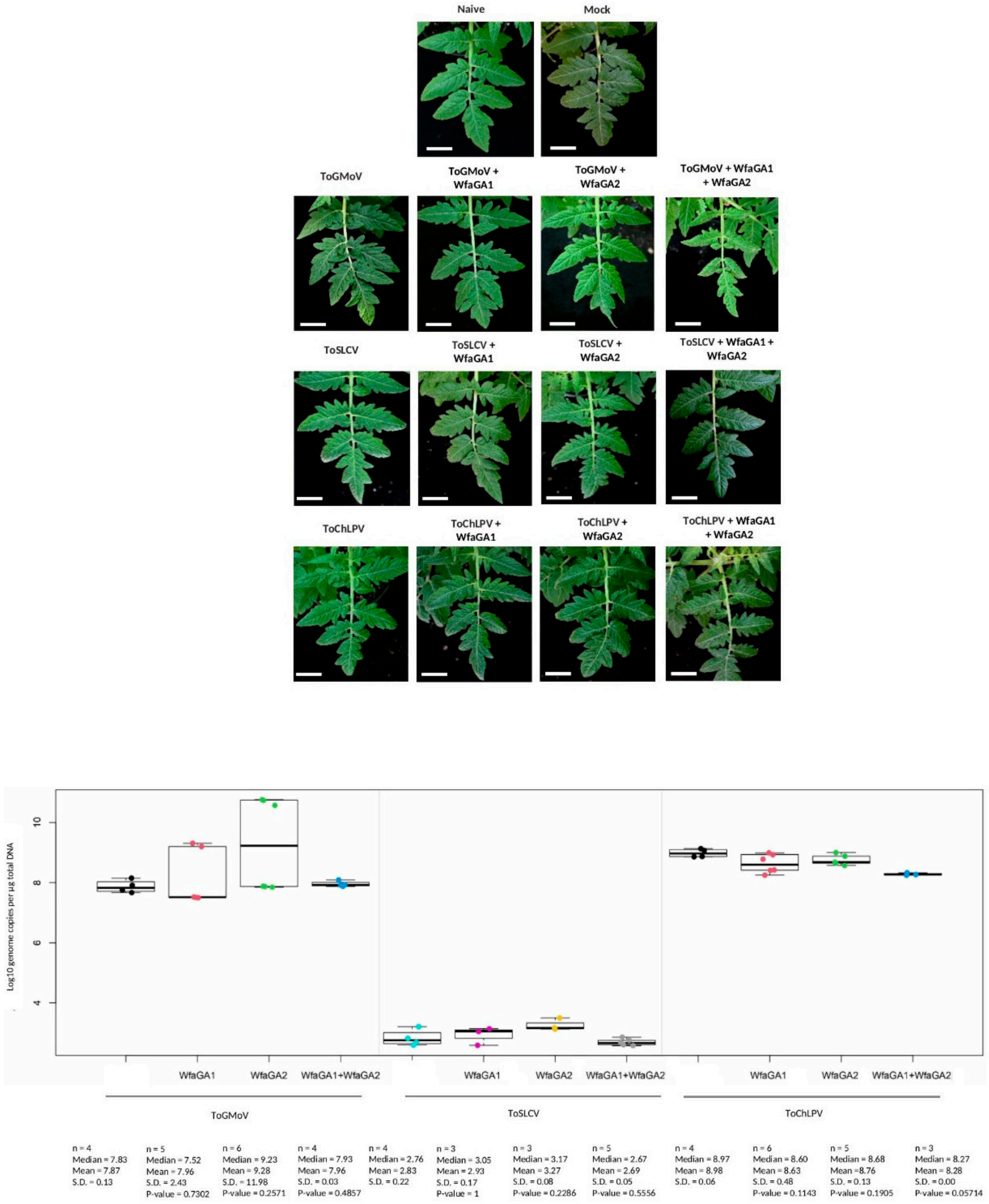

Supplementary Figure S5. Bioassay to evaluate the interaction between alphasatellites and begomoviruses. Co-inoculation of begomoviruses (ToGMoV, ToSLCV or ToChLPV) and alphasatellites (WfaGA1 and WfaGA2) in single and double combinations was performed. (**a**) Representative leaves sampled from agroinfected plants at 28 dpi. (**b**) Means of the absolute N° of viral genomes (log10) for the conditions of the agroinfections at 14 dpi were compared using the non-parametric Kruskal–Wallis test. ***: $p < 0.001$, **: $p < 0.01$, *: $p < 0.05$, ns: not significant. Each treatment includes the number of biological replicates (*n*), median, mean, standard deviation (s.d.), and p-value for each statistical comparison.

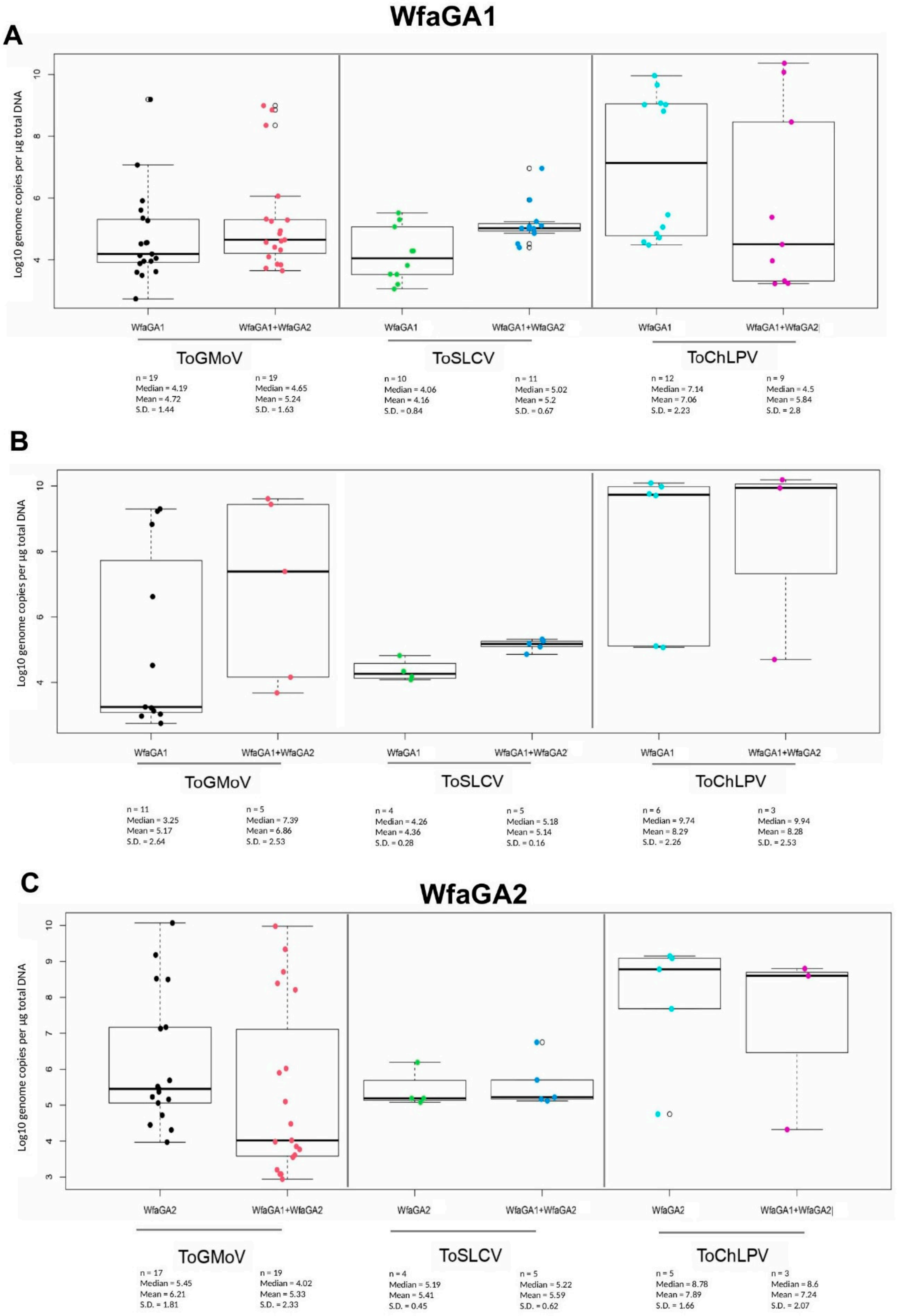

WfaGA1
A
Log10 genome copies per µg total DNA
WfaGA1
WfaGA1+WfaGA2
WfaGA1
WfaGA1+WfaGA2
WfaGA1
WfaGA1+WfaGA2
ToGMoV
ToSLCV
ToChLPV
n = 19
Median = 4.19
Mean = 4.72
S.D. = 1.44
n = 19
Median = 4.65
Mean = 5.24
S.D. = 1.63
n = 10
Median = 4.06
Mean = 4.16
S.D. = 0.84
n = 11
Median = 5.02
Mean = 5.2
S.D. = 0.67
n = 12
Median = 7.14
Mean = 7.06
S.D. = 2.23
n = 9
Median = 4.5
Mean = 5.84
S.D. = 2.8
B
Log10 genome copies per µg total DNA
WfaGA1
WfaGA1+WfaGA2
WfaGA1
WfaGA1+WfaGA2
WfaGA1
WfaGA1+WfaGA2
ToGMoV
ToSLCV
ToChLPV
n = 11
Median = 3.25
Mean = 5.17
S.D. = 2.64
n = 5
Median = 7.39
Mean = 6.86
S.D. = 2.53
n = 4
Median = 4.26
Mean = 4.36
S.D. = 0.28
n = 5
Median = 5.18
Mean = 5.14
S.D. = 0.16
n = 6
Median = 9.74
Mean = 8.29
S.D. = 2.26
n = 3
Median = 9.94
Mean = 8.28
S.D. = 2.53
C
WfaGA2
Log10 genome copies per µg total DNA
WfaGA2
WfaGA1+WfaGA2
WfaGA2
WfaGA1+WfaGA2
WfaGA2
WfaGA1+WfaGA2
ToGMoV
ToSLCV
ToChLPV
n = 17
Median = 5.45
Mean = 6.21
S.D. = 1.81
n = 19
Median = 4.02
Mean = 5.33
S.D. = 2.33
n = 4
Median = 5.19
Mean = 5.41
S.D. = 0.45
n = 5
Median = 5.22
Mean = 5.59
S.D. = 0.62
n = 5
Median = 8.78
Mean = 7.89
S.D. = 1.66
n = 3
Median = 8.6
Mean = 7.24
S.D. = 2.07

D

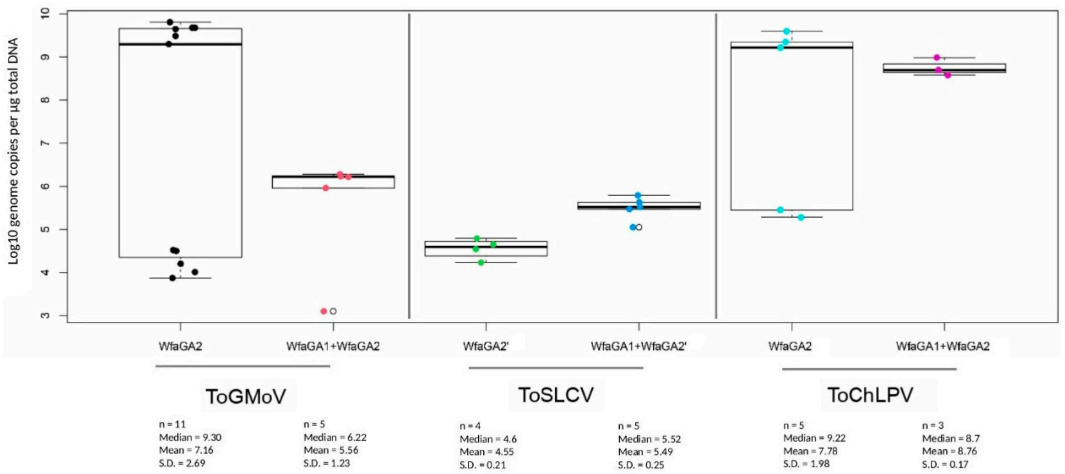

Supplementary Figure S6. Quantification of alphasatellite titers in helper virus co-infections**.** Co-inoculation of begomoviruses (ToGMoV, ToSLCV or ToChLPV) and alphasatellites (WfaGA1 and WfaGA2) in single and double combinations was performed at 14 dpi (**a and c**) and 28 dpi (**b and d**). Means of the absolute N° of viral genomes (log10) for the conditions of the agroinfections at 14 and 28 dpi dpi were compared using the non-parametric Kruskal–Wallis test. ***: $p < 0.001$, **: $p < 0.01$, *: $p < 0.05$, ns: not significant. Each treatment includes the number of biological replicates (n), median, mean, standard deviation (s.d.), and p-value for each statistical comparison.
